# Vitamin D Protects Pancreatic Cancer (PC) Cells from Death and DNA Damage Induced by Oxidative Stress

**DOI:** 10.3390/antiox14091101

**Published:** 2025-09-10

**Authors:** Izabela Szymczak-Pajor, Egle Morta Antanaviciute, Józef Drzewoski, Ireneusz Majsterek, Agnieszka Śliwińska

**Affiliations:** 1 Department of Nucleic Acid Biochemistry, Medical University of Lodz, 251 Pomorska Str., 92-213 Lodz, Poland; agnieszka.sliwinska@umed.lodz.pl; 2 Centre for Cellular Microenvironments, Mazumdar-Shaw Advanced Research Centre, University of Glasgow, Glasgow G12 8QQ, UK; 2145539a@student.gla.ac.uk; 3 Central Teaching Hospital of the Medical University of Lodz, 251 Pomorska Str., 92-213 Lodz, Poland; jozef.drzewoski@umed.lodz.pl; 4 Department of Clinical Chemistry and Biochemistry, Medical University of Lodz, 5 Mazowiecka Str., 92-215 Lodz, Poland; ireneusz.majsterek@umed.lodz.pl

**Keywords:** vitamin D_3_, oxidative stress, DNA damage, antioxidant defense enzymes, pancreatic cancer

## Abstract

In addition to its well-recognized roles in immunomodulation and calcium phosphate homeostasis, growing evidence shows that Vitamin D (Vit. D) presents a wide range of other properties, including antioxidant and anticancer effects. However, the action of Vit. D is not fully recognized in pancreatic cancer (PC) cells exposed to oxidative stress. Therefore, the aim of the present study was to investigate whether vitamin D_3_ (Vit. D_3_) protects PC cells from death induced by oxidative stress. PC cells are suggested to be resistant to oxidative stress since they demonstrate overexpression of superoxide dismutase (SOD) 1–3. The study measured PC cell viability, DNA damage level, the mRNA and protein expression of antioxidant enzymes, reactive oxygen species (ROS) level and activity of antioxidant enzymes after exposure to H_2_O_2_, Vit. D_3_ and their combinations. N-Acetyl-L-Cysteine (NAC), a well-known direct ROS scavenger, was used as a positive control. Vit. D_3_ exposure alone had no effect on PC cell viability, ROS level and DNA damage. Its impact on the mRNA and protein expression of antioxidant enzymes was also scarce. However, Vit. D_3_ protected PC cells against H_2_O_2_-induced death, similarly to NAC. It also diminished the increase in ROS and DNA damage caused by H_2_O_2_. In addition, Vit. D_3_ enhanced the mRNA expression of catalase (CAT), SOD 1–3 and glutathione peroxidase (Gpx)3, but did not affect their protein levels in PC cells exposed to oxidative stress. Interestingly, Vit. D_3_ increased CAT activity after 24 h in 1.2B4 cells and elevated the activity of both CAT and Gpx after 2 h in PANC-1 cells, which could contribute to the observed reduction of H_2_O_2_-induced ROS level. To conclude, our findings show that antioxidant properties of Vit. D_3_ may protect PC cells from oxidative stress-induced death. Therefore, further studies are needed to understand the action of Vit. D_3_ in PC cells.

## 1. Introduction

Pancreatic cancer (PC) is characterized by its high aggressiveness, mortality and resistance to drugs, especially those acting via DNA damage-induced apoptosis [1,2,3,4]. Recent epidemiological data indicate that the median survival for PC patients is approximately 4 months, with a 5-year survival rate of only 13%. The poor prognosis for PC is compounded by late-stage diagnosis. Due to the limited effectiveness of current treatments, there is a continued focus on supplementing standard therapy with novel anticancer substances [4,5].

Calcitriol, an active form of Vitamin D (Vit. D), binds to the cytosolic vitamin D receptor (VDR). The VDR then interacts with the retinoid X receptor (RXR), leading to the regulation of gene expression through Vit. D response elements (VDRE) [6,7,8,9,10,11]. This genomic action controls numerous processes, including calcium phosphate metabolism, proliferation, differentiation, angiogenesis, and immunomodulation. In addition, Vit. D exerts its non-genomic action by binding to membrane VDR, and initiating signal transduction via numerous cell signaling pathways [12,13] such as Ca^2+^/calmodulin-dependent protein kinase (PKCaMII) and mitogen-activated protein kinases (MAPKs). Thus, Vit. D exhibits a broad biological effect, regulating numerous intracellular processes and the functioning of various systems and organs [11,14,15,16,17,18,19].

PC cells demonstrate increased NADPH oxidase activity, which generates additional reactive oxygen species (ROS) [20,21]. An excess of ROS is harmful because these highly reactive molecules can damage cellular macromolecules such as lipids and DNA. Moreover, ROS can trigger the production of several toxic and highly mutagenic metabolites such as 4-hydroxy-2-nonenal (4-HNE) and malonyldialdehyde (MDA) which promote the transformation of cells into a malignant phenotype. If unrepaired, ROS-induced DNA damage leads to genomic instability and tumorigenesis. In addition, ROS promote molecular pathways (i.e., ERK1/2, NF-ĸB, c-SRC, PIK3/AKT, MMP, and RHO-RAC) are involved in tumor aggressiveness and progression by affecting the regulation of apoptosis, In addition, ROS promote molecular pathways (i.e., ERK1/2, NF-ĸB, c-SRC, PIK3/AKT, MMP, and RHO-RAC) involved in tumor aggressiveness and progression by affecting the regulation of apoptosis, and the proliferation and invasion of tumor cells [22]. Elevated ROS production in PC cells contributes to their enhanced metabolic adaptation, proliferation, survival and angiogenic potential as well as providing protection against apoptosis [23,24]. Vaquero et al. proposed that PC is so aggressive and poorly responsive to treatment due to its resistance to apoptosis [25]. To protect against the damaging effects of ROS, cells have evolved a balanced system that utilizes both enzymatic and non-enzymatic antioxidants. These endogenous and exogenous antioxidants prevent and repair damage caused by ROS, thereby decreasing the risk of numerous diseases, including cancer [26]. The most important intracellular antioxidant defenses comprise the enzymes catalase (CAT), superoxide dismutase (SOD) and glutathione peroxidase (Gpx) [27] which are known as the first-line antioxidant defense [28]. SOD includes three isoforms in mammals: cytoplasmic Cu/ZnSOD (SOD1), mitochondrial matrix MnSOD (SOD2) and extracellular Cu/ZnSOD (SOD3) [29]. The primary function of SOD is the dismutation of the superoxide radical or singlet oxygen—species generated during tissue metabolism—into H_2_O_2_ and O_2_. The accumulation of H_2_O_2_ is toxic to cells and tissues because it is rapidly converted to harmful hydroxyl radicals via the Fenton reaction in the presence of transitional metals such as Fe^2+^. To prevent this, CAT, which is abundant in peroxisomes, breaks H_2_O_2_ down into water and molecular oxygen, thereby limiting free radical-induced damage. In the mitochondria, where CAT is not present, Gpx is responsible for reducing H_2_O_2_ to water and lipid peroxides to their respective alcohols [30]. Insufficient neutralization of ROS or unrepaired cellular damage results in oxidative stress [26,31]. Oxidative stress is considered to be one of the major risk factors for cancer, including PC [32,33]. Therefore, maintaining redox homeostasis is essential to prevent the initiation of oxidative stress and the development of related diseases.

A growing body of evidence indicates that Vit. D has anticancer potential [34]. Although the effect of Vit. D on the efficacy of PC therapy has been investigated, clinical trials have shown that Vit. D or its analogs do not significantly improve the effectiveness of current anticancer treatments for PC [35,36]. The mechanisms of Vit. D’s action in cancer cells are still poorly understood, particularly in PC where recent studies have shown that cells overexpress the VDR [37]. Through its genomic activity, Vit. D regulates the expression of multiple target genes leading to a wide range of anticancer effects in various malignant cells, including anti-proliferation, anti-inflammation, induction of apoptosis, stimulation of differentiation and the inhibition of invasion, metastasis and angiogenesis [38]. Hummel et al. have suggested that the Vit. D system is deregulated in pancreatic diseases. While the levels of CYP24A1—an enzyme responsible for calcitriol degradation—are decreased in the endocrine islets during PC development, the CYP24A1 protein is accumulated in pancreatic ducts during malignant transformation. Furthermore, tumors with CYP24A1 overexpression are highly proliferative [39]. Research indicates that Vit. D has an antioxidative effect [40,41,42,43]. In turn, PC cells also exhibit overexpression of SOD1, SOD2 and SOD3, as well as lowered superoxide levels and increased H_2_O_2_ levels [44]. The antioxidant defense system is a key player in sustaining redox balance and preventing the accumulation of oxidative damage, including DNA damage. Given the broad range of Vit. D’s cellular actions and the resistance of PC cells to damaging factors (i.e., H_2_O_2_), investigating the influence of Vit. D on DNA damage and antioxidant defense in PC cells could significantly contribute to understanding of the mechanisms of its high aggressiveness and treatment resistance. The aim of the present study was to determine if Vit. D_3_ protects PC cells from death induced by oxidative stress. To achieve this goal, PC cells were exposed to H_2_O_2_ in the presence and absence of Vit. D_3_. We then measured cell survival, ROS level, DNA damage level and the activity, as well as gene and protein expression, of antioxidant enzymes.

## 2. Materials and Methods

### 2.1. Cell Culture and Treatment

The 1.2B4 cell line is a human hybrid cell line obtained by the fusion of a human pancreatic carcinoma cell line (HuP-T3) with a primary culture of human pancreatic islets. The cells were purchased from the European Collection of Authenticated Cell Cultures (ECACC, Salisbury, UK). PANC-1 (a pancreatic duct epithelioid carcinoma cell line) was purchased from the American Type Culture Collection (ATCC, Manassas, VA, USA). Both 1.2B4 and PANC-1 cells were cultured as a monolayer in the standard conditions: 37 °C, 95% air and 5% CO_2_ and 100% humidity. 1.2B4 cells were grown in RPMI 1640 supplemented with 10% fetal calf serum and 50 IU/mL Penicillin/Streptomycin (Gibco, Life Technologies, Carlsbad, CA, USA). PANC-1 cells were cultured in Dulbecco’s Modified Eagle’s Medium (DMEM) supplemented with 10% fetal bovine serum and 50 IU/mL Penicillin/Streptomycin (Gibco, Life Technologies, Carlsbad, CA, USA). The cells were used for experiments while in the logarithmic growth phase, after the third to fifth passage. Trypsin-EDTA solution was used to detach the cells. The living cells were counted using trypan blue staining.

Vit. D_3_, hydrogen peroxide (H_2_O_2_) and N-Acetyl-L-Cysteine (NAC) were purchased from Sigma Aldrich (Saint Louis, MO, USA). NAC is a known antioxidant (a free radical scavenger) and was employed as a positive control. A stock solution of Vit. D_3_ was prepared in 96% ethanol. NAC was diluted in ultrapure water. The cells were exposed to Vit. D_3_ and H_2_O_2_; a combination of Vit. D_3_ with H_2_O_2_ and NAC; and a combination of NAC with H_2_O_2_ for 24, 48 and 72 h. As a vehicle, control cells were treated with ethanol. Ethanol 1 (Et-OH_1_) corresponds to 75 nM Vit. D_3_, whereas Ethanol 2 (Et-OH_2_) corresponds to 100 nM Vit. D_3_.

### 2.2. Cell Viability Determination—MTT Assay

The impact of the tested compounds on the viability of PANC-1 and 1.2B4 cells was evaluated by the MTT [3-(4,5 dimethylthiazol-2-yl)-2,5-diphenyltetrazolium bromide] assay. PANC-1 and 1.2B4 cells were seeded in 96-well plates at a density of 2500 cells per well. After an overnight incubation, the cells achieved a logarithmic growth phase. Next, the tested compounds were added for 24, 48 and 72 h. The following concentrations were employed: Vit. D_3_ (5–100 nM), H_2_O_2_ (50–750 µM), NAC (0.001–30 mM), a combination of Vit. D_3_ (5–100 nM) with H_2_O_2_ as well as combination of NAC (0.001–30 mM) with H_2_O_2_. After the completion of exposure, 20 µL of MTT solution (5 g/L) was added to each well for an additional four-hour incubation. Subsequently, the medium was removed and 100 µL dimethyl sulfoxide (DMSO) was added to each well. The absorbance of the dissolved formazan crystals was measured at 570 nm using a SpectrostarNano microplate reader (LMG Biotech, Ortenberg, Germany). The cell viability was indicated as a percentage of control values and calculated from the mean value of three independent experiments. The results for the control group (non-treated cells) were considered as 100%.

Based on the determined viability of the tested PC cells, the following concentrations of the test compounds were used for further experiments: 75 nM and 100 nM Vit. D_3_ and 3 mM NAC for 1.2B4 and PANC-1 cells; 400 µM H_2_O_2_ for 1.2B4 cells; 300 µM H_2_O_2_ for PANC-1 cells.

### 2.3. Evaluation of DNA Damage—The Alkaline Comet Assay

One result of oxidative stress is an increase in the level of DNA damage that was detected by the alkaline version of the comet assay, performed according to Singh et al. [45] with some modifications [46,47]. The alkaline conditions are required to measure the alkaline labile sites and single and double strand breaks. PANC-1 and 1.2B4 cells were seeded in 24-well plates at a density of 10^5^ cells per well.

After 24, 48 or 72 h treatment with the tested compounds, the 1.2B4 and PANC-1 cells were washed two times with cold PBS, suspended in low-melting-point agarose (0.75%) and spread into normal-melting-point agarose (0.5%) pre-coated microscope slides. Subsequently, the cells were lysed by incubation in lysis buffer at 4 °C for 1 h. The lysis buffer was composed of NaCl, 2.5 M; EDTA, 100 mM; TritonX100, 1%; and Tris, 10 mM; pH 10. After lysis, the slides were suspended in unwinding buffer containing NaOH, 30 mM, and EDTA, 1 mM, in pH > 13 at 4 °C for 20 min.

Electrophoresis was conducted at 0.73 V/cm (28 mA) for 20 min. Then, the slides were washed three times with distilled water and drained. Finally, the slides were stained with 2 mg/mL 4′.6-diamidino-2-phenylindole dihydrochloride (DAPI) under dark conditions at a temperature of 4 °C for 30 min. The comets were observed under a fluorescence microscope (Nikon, Tokyo, Japan) at a magnification of ×200 with a video camera and ultraviolet (UV1), a filter block and personal computer equipped with the LuciaComet v. 4.51 analysis software (Laboratory Imaging, Prague, Czech Republic). DNA damage was reflected as the percentage of DNA in the tail of the observed comet and measured for 50 cells in each treatment sample.

### 2.4. mRNA Expression—qRT-PCR

1.2B4 and PANC-1 cells were seeded on 12-well plates at a density of 10^6^ and incubated with the tested compounds for 24, 48 and 72 h. After completion of treatment with the tested compounds, total RNA was isolated from using a Total RNA Mini Kit (A&A Biotechnology, Gdynia, Poland). Both the quality and quantity of the isolated RNA were assessed with a Nanodrop 2000 reader (Thermofisher Scientific Inc., Waltham, MA, USA). An amount of 1 μg of total isolated RNA was taken for cDNA synthesis. The reaction of reverse transcription was conducted using a High Capacity cDNA Reverse Transcription Kit (Thermofisher Scientific Inc., Waltham, MA, USA), according to the manufacturer’s protocol. cDNA was employed to run qRT-PCR by using the TaqMan assays targeting the studied genes (ID: Hs00156308_m1 for CAT, Hs00533490_m1 for SOD1, Hs00167309_m1 for SOD2, Hs00162090_m1 for SOD3, Hs00173566_m1 for Gpx3, Hs99999905_m1 for GAPDH) (Life Technologies, Carlsbad, CA, USA) and TaqMan Universal Master Mix (Life Technologies, Carlsbad, CA, USA).

Each sample was subjected to qPCR in duplicate. The expression of the studied genes was calculated as the mean results of three independent experiments. The levels of expression of the studied genes were normalized to an endogenous control, GAPDH. Fold change was calculated by the comparative Ct (ΔΔCt) method using the following equation: Relative Quantity (RQ): (2^−ΔΔCt^) [48].

### 2.5. Protein Expression Analysis—Western Blotting

The 1.2B4 and PANC-1 cells were incubated with the tested compounds for 24, 48 and 72 h. Following this, total protein was isolated from PANC-1 and 1.2B4 cells with RIPA lysis buffer composed of 50 mM Tris-HCl, 150 mM NaCl, 1% sodium deoxycholate, 0.1% SDS and 2 mM EDTA and supplemented with a protease inhibitor cocktail (Thermofisher Scientific Inc, Waltham, MA, USA) according to the manufacturer’s instruction. Next, the concentration of protein samples was assessed by Micro BCA^TM^ Protein Assay Kit (Life Technologies, Carlsbad, CA, USA). In total, 10 µg of protein of each sample were analyzed by immunoblotting and separated by electrophoresis in denaturing polyacrylamide 4–20% Mini Protean TGX Stain-Free Gel (BioRad, Hercules, CA, USA) at 200 V and 31 mA, for 40 min.

After electrophoresis, the separated proteins were transferred from the gel to a polyvinylidene fluoride (PVDF) membrane using the Trans-Blot Turbo Transfer System (BioRad, Hercules, CA, USA). Then, the PVDF membranes were blocked in 5% nonfat milk diluted in a Tris-buffered saline [TBST] buffer with 0.1% Tween 20. After blocking, the membranes were incubated with rabbit primary anti-CAT antibody (FNab01302), anti-SOD1 antibody (FNab08103), anti-SOD2 (FNab08104) antibody, anti-SOD3 antibody (FNab08105), anti-Gpx3 (FNab03621) antibody (Fine-Test, Wuhan, China) and anti-GAPDH (ab9485) antibody (Abcam, Cambridge, UK) overnight at 4 °C. Then, the blots were washed in TBST buffer (3 times for 15 min) and incubated with anti-rabbit secondary antibodies (ab205718) (Abcam, Cambridge, UK) for 4 h at 4 °C. After washing in TBST buffer (3 times for 15 min), the bands were visualized with ECL Blotting Substrate (BioRad, Hercules, CA, USA) using ChemiDoc MP Imaging System (BioRad, Hercules, CA, USA). Densitometric analysis of visualized protein level was carried out by using Image J 1.34s software (Wayne Rasband, National Institutes of Health, Bethesda, MD, USA). GAPDH was employed as a reference protein standard. The protein expression was calculated as the mean of the value of three independent experiments.

### 2.6. Determination of ROS Level

The level of intracellular ROS production was measured directly in the cell monolayer in black 96-well flat-bottom microtiter plates with a Fluoroskan Ascent FL microplate reader (Thermo Fisher Scientific, Waltham, MA, USA). The cells were seeded at a density of 10^4^ per well and incubated with tested compounds for 2 h and 24 h after reaching the logarithmic growth phase. Then, the medium was removed and cells were incubated with 5 μM DCFH2-DA at 37 °C for 30 min. After the completion of incubation, the fluorescence of DCF was measured at 530 nm after excitation at 485 nm. Inside the cells, DCFH2-DA after deacetylation to DCFH2 is oxidized by ROS to DCF (fluorescent derivative of DCFH2). The fluorescence intensity of DCF was used to determine the level of ROS. The ROS level was calculated as the mean of value of three independent experiments.

### 2.7. Determination of Antioxidant Enzymes’ Activity

1.2B4 and PANC-1 cells were seeded on 12-well plates at a density of 10^6^ per well. After achieving the logarithmic growth phase by overnight incubation, the cells were exposed to the tested compounds for 2 h and 24 h. After the completion of incubation, the cells were collected using a rubber policeman and centrifuged. Collected cell pellets were homogenized in cold buffers that contain: 50 mM potassium phosphate, pH 7.0, containing 1 mM EDTA for CAT; 20 mM HEPES, pH 7.2, containing 1 mM EGTA, 210 mannitol and 70 mM sucrose for SOD; 50 mM Tris-HCl, pH 7.5, 5 mM EDTA and 1 mM DTT for Gpx. The obtained homogenates were centrifuged. The supernatant samples were used for determination of the enzymatic activity of antioxidant enzymes. We employed the following kits: Catalase Assay Kit, Superoxide Dysmutase Assay Kit and Glutathione Peroxidase Assay Kit (Cayman Chemical, Ann Arbor, MI, USA) to determine the enzymatic activity of CAT, SOD and Gpx.

CAT activity measurement includes the reaction of the enzyme with methanol which takes place in the presence of H_2_O_2_. The produced formaldehyde was determined colorimetrically with chromogen (4-amino-3-hydrozino-5-mercapto-1,2,4-triazole). The chromogen reacts with formaldehyde leading to formation of bicyclic heterocycle that changes from colorless to a purple color upon oxidation. The absorbance was measured at 540 nm.

The SOD activity measurement employs a tetrazolium salt for detection of superoxide radicals formed by hypoxantine and xantine oxidase. The measured change in absorbance per minute (25 °C; pH = 8.0), defined as one unit of SOD, indicates the amount of this enzyme needed to perform 50% dismutation of the superoxygen radical. The absorbance was measured at 450 nm.

The principle of the Gpx activity assay involves the conversion of oxidized glutathione (glutathione disulfide, GSSG) to its reduced form by glutathione reductase (GR) and NADPH. The oxidation of NADPH to NADP+ is related to decrease in absorbance. In turn, the decrease in absorbance measured over time is directly proportional to Gpx activity since it is rate limiting. The absorbance was measured ten times (once per minute) at 340 nm.

### 2.8. Statistical Analysis

Statistical analysis was carried out using GraphPad Prism 6.0 (San Diego, CA, USA). The differences between two groups were evaluated by using Student’s *t*-test and Mann–Whitney U-test after testing for normality. To determine the differences between three or more groups, ANOVA with Tukey’s multiple comparison test or the non-parametric Kruskal–Wallis test with Dunn’s multiple comparison test were employed, according to data distribution and homogeneity of variance. The data are presented as mean ± standard deviation (SD) from three independent experiments. The *p*-value < 0.05 was considered to be statistically significant.

## 3. Results

### 3.1. Vit. D_3_ Protects 1.2B4 and PANC-1 Cells from the Cytotoxic Effect of H_2_O_2_

**Figure 1:**
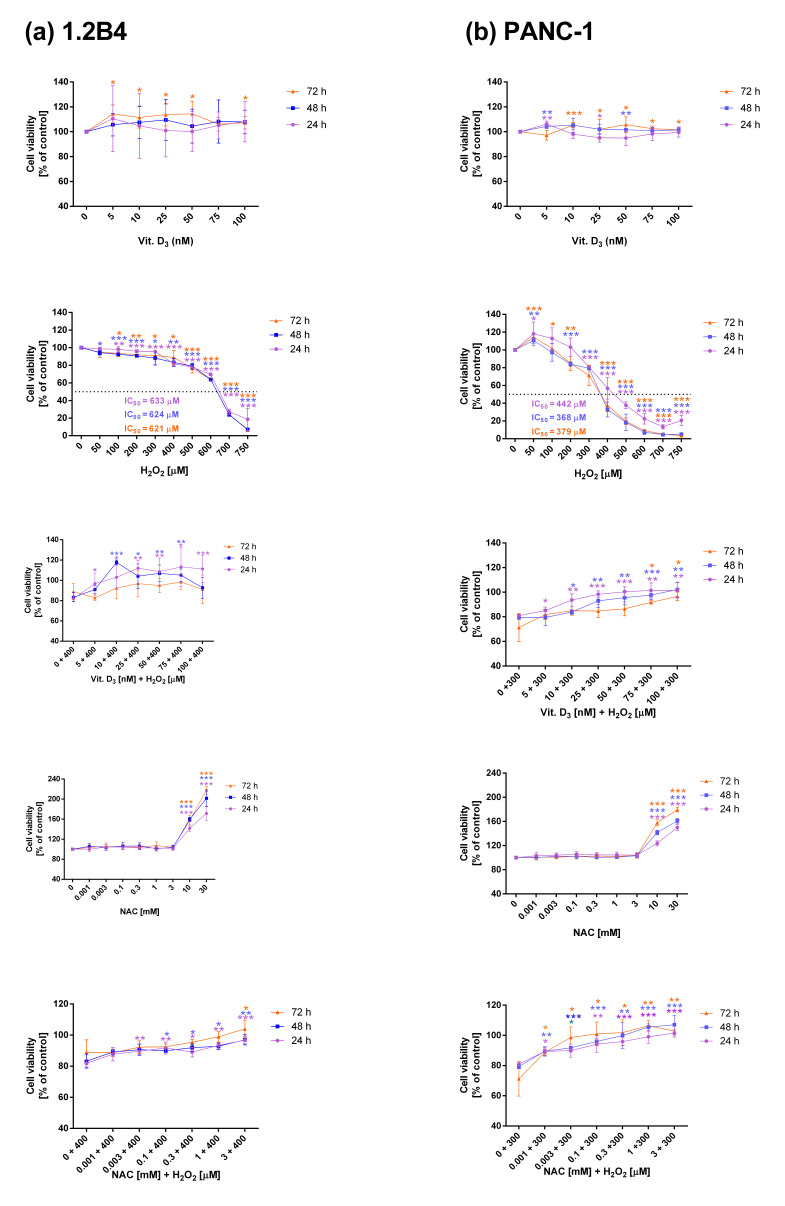
The effect of vitamin D_3_ (Vit. D_3_), hydrogen peroxide (H_2_O_2_), N-Acetyl-L-Cysteine (NAC), a combination of Vit. D_3_ with H_2_O_2_ (Vit. D_3_ + H_2_O_2_) and NAC with H_2_O_2_ (NAC + H_2_O_2_) on the viability of (**a**) 1.2B4 cells and (**b**) PANC-1 cells after 24 h (violet lines), 48 h (blue lines) and 72 h (orange lines) of exposure. Cell viability was determined by MTT assay. The PC cells were treated with Vit D_3_ (5–100 nM), H_2_O_2_ (50–750 µM), Vit. D_3_ (5–100 nM) with H_2_O_2_ (400 µM for 1.2B4 cells or 300 µM for PANC-1 cells), NAC (0.001–30 mM), NAC (0.001–30 mM) with H_2_O_2_ (400 µM for 1.2B4 cells or 300 µM for PANC-1 cells) for 24 h, 48 h and 72 h. After treatment, MTT was added for 4 h. Then, DMSO was added to dissolve formazan crystals and absorbance was measured at 570 nm with a microplate reader. The cell viability (the data) was determined as a percentage of control. The values for the control group (non-treated cells) were considered to be 100%. The data are expressed as the mean ± standard deviation (SD) of the three independent experiments. * *p* < 0.05, ** *p* < 0.01 and *** *p* < 0.001. vs. control (non-treated cells for Vit. D_3_, H_2_O_2_, NAC or H_2_O_2_–treated cells for Vit. D_3_ + H_2_O_2_, NAC + H_2_O_2_). Violet stars refer to 24 h; blue stars to 48 h; orange stars to 72 h.

The effect of Vit. D_3_, H_2_O_2_, NAC, combination of Vit. D_3_ with H_2_O_2_, and NAC with H_2_O_2_ (Figure 1) on the viability of PC cells was determined via MTT assay. In the tested concentration range, Vit. D_3_ did not affect the viability of 1.2B4 cells. In turn, H_2_O_2_ significantly reduced 1.2B4 cell viability in a dose-dependent manner. To examine how Vit. D_3_ affects H_2_O_2_-induced cell death, 1.2 B4 cells were exposed to 400 µM H_2_O_2_ and Vit. D_3_ (5–100 nM). Vit. D_3_ decreased H_2_O_2_-induced cytotoxic effect toward 1.2B4 cells in a dose-dependent manner. The lowest protective potential was demonstrated by 5 nM of Vit. D_3_. NAC did not affect 1.2B4 cell viability in the concentration range 0.001–3 mM, but it increased 1.2B4 cell viability at concentrations above 3 mM. The combination of NAC with H_2_O_2_ protected 1.2B4 cells against the cytotoxic effect of H_2_O_2_.

In case of PANC-1 cells, Vit. D_3_ also did not significantly alter cell viability. As expected, H_2_O_2_ exposure resulted in a pronounced increase in the number of dead PANC-1 cells in a dose-dependent manner. The combined exposure to 300 µM H_2_O_2_ and Vit. D_3_ reduced the cytotoxic effect of H_2_O_2_ toward PANC-1 cells. This protective effect increased with the concentration of Vit. D_3_ in all tested incubation times. NAC did not affect PANC-1 cell viability in the tested concentration range, but showed an increase in PANC-1 cell viability at concentrations above 3 mM. The combination of NAC with H_2_O_2_ protected PANC-1 cells against the cytotoxic effect of H_2_O_2_.

Taken together, high IC_50_ values for the cytotoxic H_2_O_2_ observed in both cell lines suggest the resistance of PC cells to oxidative stress. The 1.2B4 and PANC-1 cells had a similar response to H_2_O_2_, although 1.2B4 cells were less sensitive to H_2_O_2_ than PANC-1cells. In our study, we used high concentrations of H_2_O_2_ due to the resistance of PC cells to oxidative stress associated with resistance to damaging agents, cytostatics and chemotherapy.

Both cell lines responded similarly to NAC alone and Vit. D_3_ alone, as well as to combined exposure (Vit. D_3_ and H_2_O_2_, and NAC and H_2_O_2_). Thus, one can see that Vit. D_3_ exerted coherent cytoprotective effects similar to the well-known antioxidant NAC.

### 3.2. Vit. D_3_ Exerts a Protective Effect Against H_2_O_2_-Induced DNA Damage in PC Cells, but Less than NAC

**Figure 2:**
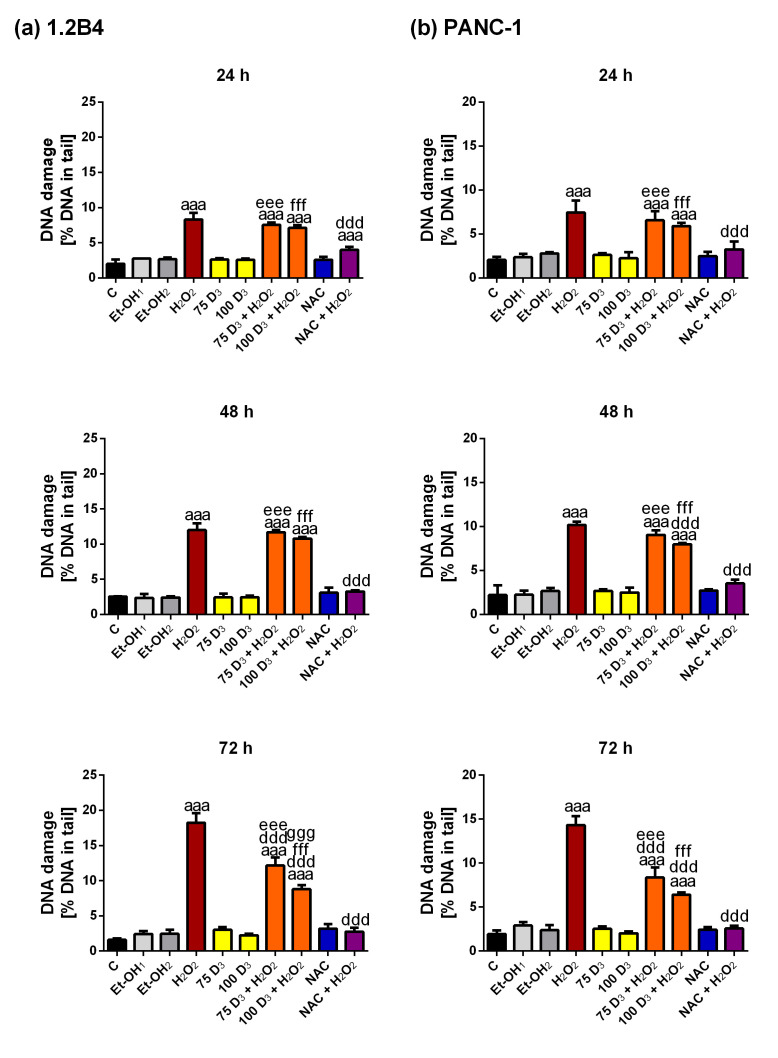
The effect of vitamin D_3_ (Vit. D_3_), hydrogen peroxide (H_2_O_2_), N-Acetyl-L-Cysteine -acetylcysteine (NAC) and the combinations of Vit. D_3_ with H_2_O_2_ (Vit. D_3_ + H_2_O_2_) and NAC with H_2_O_2_ (NAC + H_2_O_2_) on DNA damage level in pancreatic cancer (PC) cells. The 1.2B4 cells (**a**) and PANC-1 (**b**) cells were non-treated (C, black bars) and treated with ethanol (Et-OH, gray bars), Vit D_3_ (75 nM; 100 nM, yellow bars), H_2_O_2_ (400 µM for 1.2B4 cells; 300 µM for PANC-1 cells, brown bars), Vit. D_3_ (75 nM; 100 nM) with H_2_O_2_ (400 µM for 1.2B4 cells; 300 µM for PANC-1 cells, orange bars), NAC (3 mM, blue bars), NAC (3 mM) with H_2_O_2_ (400 µM for 1.2B4 cells; 300 µM for PANC-1 cells, violet bars) for 24, 48 and 72 h. The data are expressed as a % DNA in the tail of the comet counted from 50 cells. The data are presented as the mean ± standard deviation (SD) of the three independent experiments. ^aaa^ *p* < 0.001 vs. C; ^ddd^ *p* < 0.001 vs. H_2_O_2_; ^eee^ *p* < 0.001 vs. 75D_3_; ^fff^ *p* < 0.001 vs. 100D_3_; ^ggg^ *p* < 0.01 vs. 75D_3_ + H_2_O_2_.

H_2_O_2_ is a well-known inducer of oxidative stress that evokes various forms of DNA damage such as oxidative modifications to bases, as well as single- and double-strand breaks and alkaline-labile sites. Therefore, the next stage of the study examined how Vit. D_3_ influences the DNA damage induced by H_2_O_2_. The representative photos are given in Supplementary Figure S3. The 1.2B4 (Figure 2a) and PANC-1 (Figure 2b) cells were exposed to Vit. D_3_, H_2_O_2_, Vit. D_3_ combined with H_2_O_2_ and NAC combined with H_2_O_2_. The resulting level of DNA damage was evaluated by alkaline comet assay. As expected, H_2_O_2_ evoked a time-dependent increase in the level of DNA damage in 1.2B4 and PANC-1 cells. In contrast, in both cell lines, exposure to Vit. D_3_ and NAC alone did not elevate DNA damage. Vit. D_3_ slightly reduced the level of H_2_O_2_-induced DNA damage, but a significant reduction was observed only after 72 h in both cell lines. Moreover, the decrease of H_2_O_2_-evoked DNA damage by NAC was pronouncedly higher than that detected for Vit. D_3_ and observed for 24, 48 and 72 h of exposure. To conclude, although Vit. D_3_ was able to diminish the level of DNA damage caused by oxidative stress in both PC cell lines, its actions seemed to be less intensive than those of NAC. This finding suggests that Vit. D_3_ and NAC may act through different mechanisms. It is possible that NAC, being a free radical scavenger, removes ROS and thereby prevents DNA from damage evoked by H_2_O_2_ earlier [49].

### 3.3. Vit. D_3_ Is More Effective than NAC at Increasing the mRNA Expression of Antioxidant Enzymes Lowered by H_2_O_2_ in PC Cells

**Figure 3:**
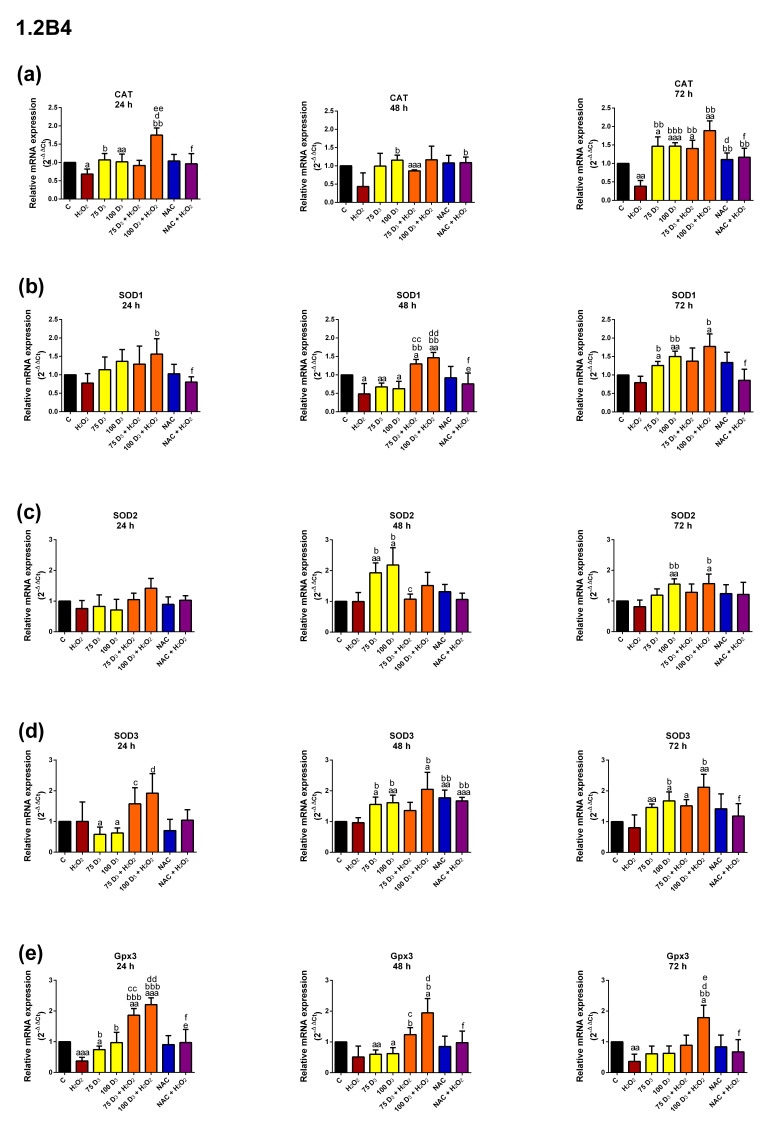
The effect of vitamin D_3_ (Vit. D_3_), hydrogen peroxide (H_2_O_2_), N-Acetyl-L-Cysteine (NAC), the combination of Vit. D_3_ with H_2_O_2_ (Vit. D_3_ + H_2_O_2_) and the combination of NAC with H_2_O_2_ (NAC + H_2_O_2_) on mRNA expression of the antioxidant enzymes (**a**) catalase (CAT), (**b**) sodium dismutase 1 (SOD1), (**c**) sodium dismutase 2 (SOD2), (**d**) sodium dismutase 3 (SOD3) and (**e**) glutathione peroxidase 3 (Gpx3) in 1.2B4 cells. The 1.2B4 cells were non-treated (C, black bars) and treated with Vit D_3_ (75 nM; 100 nM, yellow bars), H_2_O_2_ (400 µM, brown bars), Vit. D_3_ (75 nM; 100 nM) with H_2_O_2_ (400 µM, orange bars), NAC (3 mM, blue bars), NAC (3 mM) with H_2_O_2_ (400 µM, violet bars) for 24, 48 and 72 h. Data are expressed as the mean ± standard deviation (SD) of the fold change of three independent experiments in relation to the untreated control. GAPDH was used as a reference gene. ^a^ *p* < 0.05; ^aa^ *p* < 0.01; ^aaa^ *p* < 0.001 vs. C; ^b^ *p* < 0.05; ^bb^ *p* < 0.01; ^bbb^ *p* < 0.001 vs. H_2_O_2_; ^c^ *p* < 0.05; ^cc^ *p* < 0.01 vs. 75D_3_; ^d^ *p* < 0.05; ^dd^ *p* < 0.01 vs. 100D_3_; ^e^ *p* < 0.05; ^ee^ *p* < 0.01 vs. 75D_3_ + H_2_O_2_; ^f^ *p* < 0.05 vs. 100D_3_ + H_2_O_2_.

**Figure 4:**
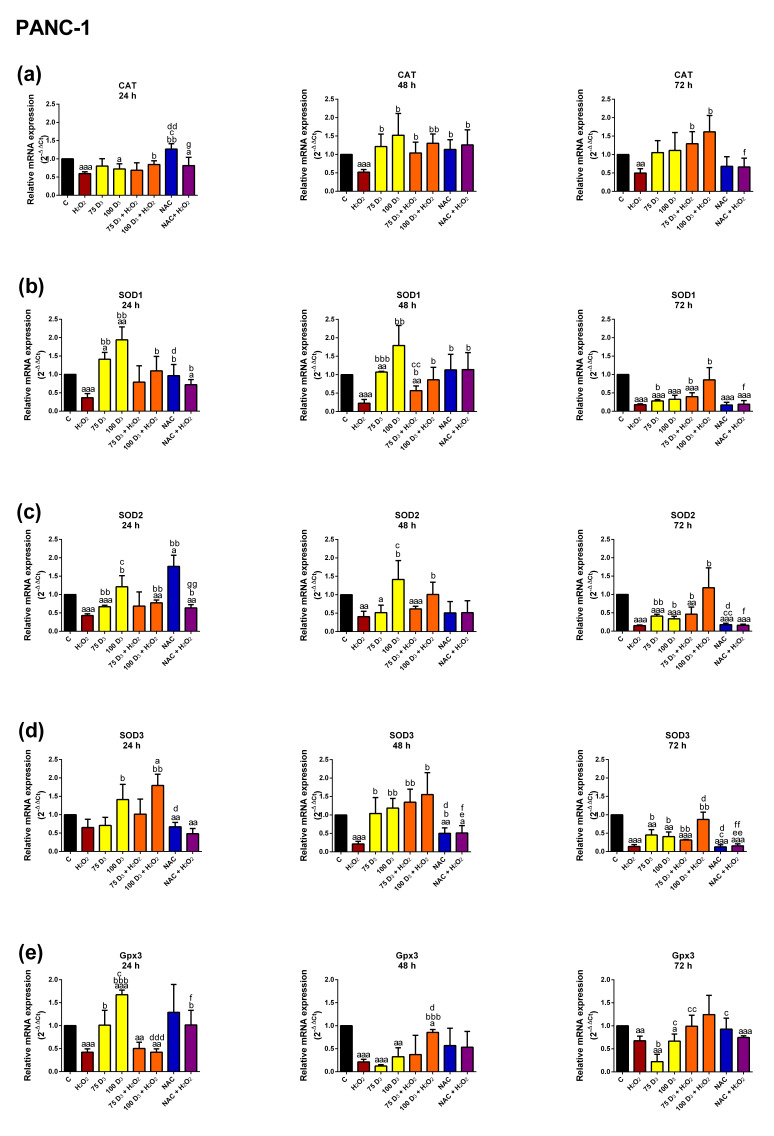
The effect of vitamin D_3_ (Vit. D_3_), hydrogen peroxide (H_2_O_2_), N-Acetyl-L-Cysteine (NAC), the combination of Vit. D_3_ with H_2_O_2_ (Vit. D_3_ + H_2_O_2_) and the combination of NAC with H_2_O_2_ (NAC + H_2_O_2_) on the mRNA expression of the antioxidant enzymes (**a**) catalase (CAT), (**b**) sodium dismutase 1 (SOD1), (**c**) sodium dismutase 2 (SOD2), (**d**) sodium dismutase 3 (SOD3), (**e**) glutathione peroxidase 3 (Gpx3) in PANC-1 cells. The PANC-1 cells were non-treated (C, black bars) and treated with Vit D_3_ (75 nM; 100 nM, yellow bars), H_2_O_2_ (300 µM, brown bars), Vit. D_3_ (75 nM; 100 nM) with H_2_O_2_ (300 µM, orange bars), NAC (3 mM, blue bars) and NAC (3 mM) with H_2_O_2_ (300, violet bars) for 24, 48 and 72 h. Data are expressed as the mean ± standard deviation (SD) of the fold change of three independent experiments in relation to the untreated control. GAPDH was used as a reference gene. ^a^ *p* < 0.05; ^aa^ *p* < 0.01; ^aaa^ *p* < 0.001 vs. C; ^b^ *p* < 0.05; ^bb^ *p* < 0.01; ^bbb^ *p* < 0.001 vs. H_2_O_2_; ^c^ *p* < 0.05; ^cc^ *p* < 0.01 vs. 75D_3_; ^d^ *p* < 0.05; ^dd^ *p* < 0.01; ^ddd^ *p* < 0.001 vs. 100D_3_; ^e^ *p* < 0.05; ^ee^ *p* < 0.01 vs. 75D_3_ + H_2_O_2_; ^f^ *p* < 0.05; ^ff^ *p* < 0.01 vs. 100D_3_ + H_2_O_2_; ^g^ *p* < 0.05; ^gg^ *p* < 0.01 vs. NAC.

Since Vit. D_3_ does not directly act on ROS level, the study examined its action on the expression of the *CAT, SOD1, SOD2, SOD3* and *Gpx3* antioxidant defense system genes. The effects of Vit. D_3_, H_2_O_2_, NAC, Vit. D_3_ combined with H_2_O_2_ and NAC combined with H_2_O_2_ on mRNA expression, as determined by RT-qPCR, are shown in Figure 3 (for 1.2B4 cells) and in Figure 4 (for PANC-1 cells).

In the 1.2B4 cells, H_2_O_2_ treatment resulted in a time-dependent decrease in *CAT* mRNA expression. Exposure to Vit. D_3_ increased *CAT* expression, but markedly only after 24 and 72 h. Treatment with Vit. D_3_ + H_2_O_2_ increased *CAT* expression in comparison to H_2_O_2_ exposure alone. Higher upregulation was evoked by 100 nM Vit. D_3_, especially after 24 and 72 h. In turn, exposure of 1.2 B4 cells to the combination of NAC and H_2_O_2_ significantly upregulated *CAT* mRNA expression after 48 and 72 h.

H_2_O_2_ had a slight influence on *SOD1* expression, causing a significant downregulation only after 48 h. Exposure to Vit. D_3_ reduced SOD1 expression after 48 h but increased it after 72 h. Treatment with Vit. D_3_ + H_2_O_2_ resulted in higher *SOD1* expression compared to H_2_O_2_ alone, especially for 100 nM Vit. D_3_.

*SOD2* expression was not altered by H_2_O_2_ treatment. Vit. D_3_ significantly upregulated *SOD2* expression after 48 and 72 h in a dose-dependent manner, with a more pronounced effect observed after 48 h. Treatment with 100 nM Vit. D_3_ and H_2_O_2_ significantly increased *SOD2* expression compared to H_2_O_2_ alone, but only after 72 h.

*SOD3* expression did not change after exposure to H_2_O_2_. Vit. D_3_ treatment decreased *SOD3* expression after 24 h, but a dose-dependent increase was reported after 48 and 72 h. In turn, 48 h NAC exposure evoked a significant elevation in *SOD3* expression. The 100 nM Vit. D_3_ + H_2_O_2_ treatment pronouncedly increased *SOD3* expression compared to H_2_O_2_ after 48 and 72 h. The NAC + H_2_O_2_ treatment markedly increased *SOD3* expression in relation to H_2_O_2_ after 48 h.

*Gpx3* expression was reduced after 24 and 72 h treatment with H_2_O_2_. Exposure to 75 nM Vit. D_3_ significantly reduced *Gpx3* expression after 24 h, while both doses of Vit. D_3_ markedly downregulated it after 48 h. Incubation with Vit. D_3_ + H_2_O_2_ caused a pronounced increase in *Gpx3* expression compared to H_2_O_2_ alone; this effect was marked after 72 h at the higher dose of Vit. D_3_ (100 nM).

In PANC-1 cells, H_2_O_2_ exposure decreased *CAT* expression at 24, 48 and 72 h. Vit. D_3_ treatment did not affect *CAT* expression. Treatment with Vit. D_3_ + H_2_O_2_ increased *CAT* expression; however, the elevation was more pronounced for 100 nM Vit. D_3_ as compared to H_2_O_2_. Exposure to NAC and H_2_O_2_ increased *CAT* expression in relation to H_2_O_2_ alone after 48 h.

Similarly to *CAT* expression, H_2_O_2_ evoked a decrease in *SOD1* expression at all studied time points in the PANC-1 cells. Vit. D_3_ increased *SOD1* expression in a dose-dependent manner after 24 h and 48 h, but decreased it after 72 h. NAC significantly lowered *SOD1* expression after 72 h. Exposure to Vit. D_3_ + H_2_O_2_ resulted in a dose-dependent *SOD1* mRNA increase in relation to H_2_O_2_ alone. NAC and H_2_O_2_ treatment increased *SOD1* expression after 24 and 48 h compared to H_2_O_2_ alone.

H_2_O_2_ significantly decreased *SOD2* expression after 24, 48 and 72 h. Vit. D_3_ had varied effects on *SOD2* expression depending on its concentration and incubation periods: 75 nM Vit. D_3_ decreased *SOD2* expression at all time points, while 100 nM Vit. D_3_ lowered expression after 72 h. NAC upregulated *SOD2* expression after 24 h but downregulated it after 72 h. Treatment with 100 nM Vit. D_3_ and H_2_O_2_ increased *SOD2* expression at all time points compared to H_2_O_2_ alone. NAC + H_2_O_2_ treatment increased *SOD2* expression compared to H_2_O_2_ alone but only after 24 h.

H_2_O_2_ treatment resulted in a time-dependent decrease in *SOD3* expression. Both Vit. D_3_ concentrations decreased expression of *SOD3* after 72 h. NAC treatment lowered *SOD3* expression at all time points. Vit. D_3_ + H_2_O_2_ increased *SOD3* expression compared to H_2_O_2_ exposure after 24, 48 and 72 h. Treatment with NAC + H_2_O_2_ did not significantly alter *SOD3* expression compared to H_2_O_2_ alone.

As before, H_2_O_2_ lowered *Gpx3* expression at all studied time points. Incubation with 100 nM Vit. D_3_ increased *Gpx3* expression after 24 h. Both Vit. D_3_ concentrations diminished significantly *Gpx3* expression after 48 and 72 h. Treatment with 100 nM Vit. D_3_ + H_2_O_2_ evoked a significant increase in *Gpx3* expression compared to H_2_O_2_ alone for 48 h. NAC + H_2_O_2_ significantly increased *Gpx3* expression compared to H_2_O_2_, but only after 24 h.

In conclusion, H_2_O_2_ decreased the expression of most examined antioxidant enzyme genes in both the 1.2B4 and PANC-1 cells. In the 1.2B4 cells, exposure to Vit. D_3_ alone slightly increased the expression of *CAT* and *SOD1–3,* but did not alter *Gpx3* expression. In turn, in PANC-1 cells weak influence of Vit. D_3_ on the mRNA expression of antioxidant enzymes was not unidirectional, as we observed both down- and upregulation of particular isoforms. In 1.2B4 cells NAC exposure did not affect the mRNA expression of antioxidant enzymes; however, in PANC-1 cells its effect was diverse. Thus, one can notice that there is a difference in the mRNA expression of antioxidant enzymes in response to the Vit. D_3_ alone or NAC alone treatments between 1.2B4 and PANC-1 cells. As is further underlined by the differences in response to NAC + H_2_O_2_ in relation to H_2_O_2_. Namely in PANC-1 cells treated with NAC + H_2_O_2_, the mRNA expression of the majority of the antioxidant enzymes slightly upregulated, in contrast to 1.2B4 cells. Both PC cell lines exposed to H_2_O_2_ and Vit. D_3_ exhibited the significantly upregulated expression of antioxidant enzymes, indicating that Vit. D_3_ may have antioxidant potential under oxidative stress conditions.

### 3.4. Vit. D_3_ Elevates CAT Protein Expression During H_2_O_2_-Induced Oxidative Stress in 1.2B4 Cells

To further explore the antioxidant action of Vit. D_3_, the next part of the study examined its effect on the protein expression of CAT, SOD1, SOD2, SOD3 and Gpx3. The effects of treatment with Vit. D_3_, H_2_O_2_, NAC and combinations of Vit. D_3_ with H_2_O_2_ and NAC with H_2_O_2_ on protein expression are given in Supplementary Figure S1 (for 1.2B4 cells) and in Supplementary Figure S2 (for PANC-1 cells); the data was obtained by Western blotting.

The impact of the tested compounds alone and in combinations on protein levels of antioxidant enzymes was slight. In 1.2B4 cells we observed an increase only in CAT protein expression after exposure to 75 nM Vit. D_3_ and H_2_O_2_ after 72 h. A pronounced increase in SOD3 protein expression was detected after exposure to 75 nM Vit. D_3_ for 48 h. None of the applied treatments affected the protein expression of SOD1, SOD2 and Gpx3 in 1.2B4 cells.

In the case of PANC-1 cells, only NAC with H_2_O_2_ significantly increased CAT protein expression compared to H_2_O_2_ after 24 h. Exposure to NAC alone markedly upregulated the protein expression of Gpx3 protein after 24 h. To conclude, Vit. D_3_ alone, or in the presence of oxidative stress, did not significantly affect the level of CAT, SOD1, SOD2, SOD3 or Gpx3 in either 1.2B4 or PANC-1 cells.

### 3.5. Vit. D_3_ Increases Activity of CAT After 24 h in 1.2B4 Cells and Elevates Activity of CAT and Gpx After 2 h in PANC-1 Cells Leading to Reduction of H_2_O_2_-Induced Level of ROS

**Figure 5:**
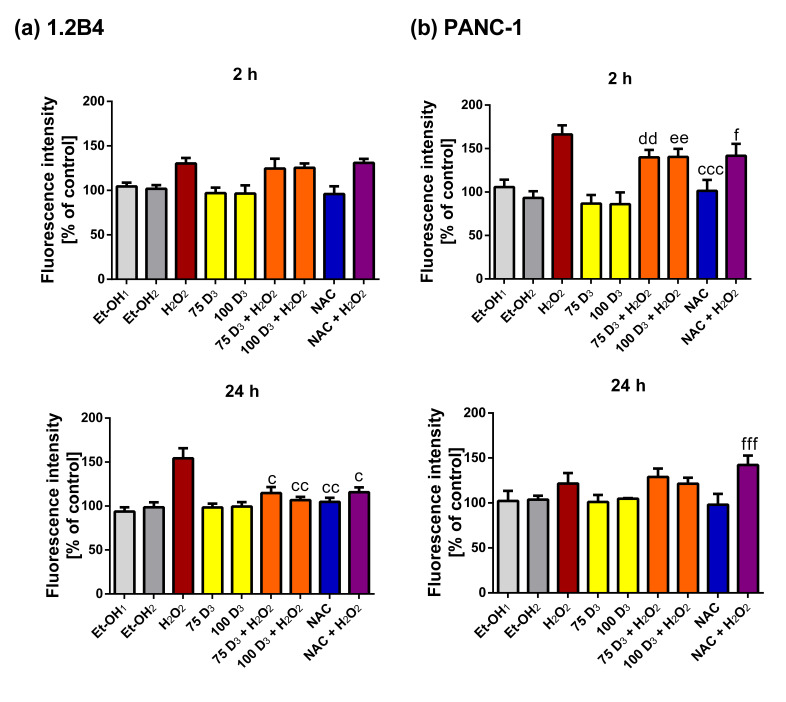
The effect of vitamin D_3_ (Vit. D_3_), hydrogen peroxide (H_2_O_2_), N-Acetyl-L-Cysteine (NAC), the combination of Vit. D_3_ with H_2_O_2_ (Vit. D_3_ + H_2_O_2_) and the combination of NAC with H_2_O_2_ (NAC + H_2_O_2_) on the reactive oxygen species (ROS) level in pancreatic cancer (PC) cells. The 1.2B4 cells (**a**) and PANC-1 (**b**) cells were treated with ethanol (Et-OH, gray bars), Vit D_3_ (75 nM; 100 nM, yellow bars), H_2_O_2_ (400 µM for 1.2B4 cells; 300 µM for PANC-1 cells, brown bars), Vit. D_3_ (75 nM; 100 nM) with H_2_O_2_ (400 µM for 1.2B4 cells; 300 µM for PANC-1 cells, orange bars), NAC (3 mM, blue bars) and NAC (3 mM) with H_2_O_2_ (400 µM for 1.2B4 cells; 300 µM for PANC-1 cells, violet bars) for 2 and 24 h. After the completion of treatment, DCFH2-DA was added and incubated for 30 min. Then, the fluorescence of DCF was measured using microplate reader at a 530 nm excitation at 485 nm. The data are expressed as a percentage of the control. The data are presented as the mean ± standard deviation (SD) of the three independent experiments. ^c^ *p* < 0.05; ^cc^ *p* < 0.01; ^ccc^ *p* < 0.001 vs. H_2_O_2_; ^dd^ *p* < 0.01 vs. 75D_3_; ^ee^ *p* < 0.01 vs. 100D_3_; ^f^ *p* < 0.05; ^fff^ *p* < 0.001 vs. NAC.

**Figure 6:**
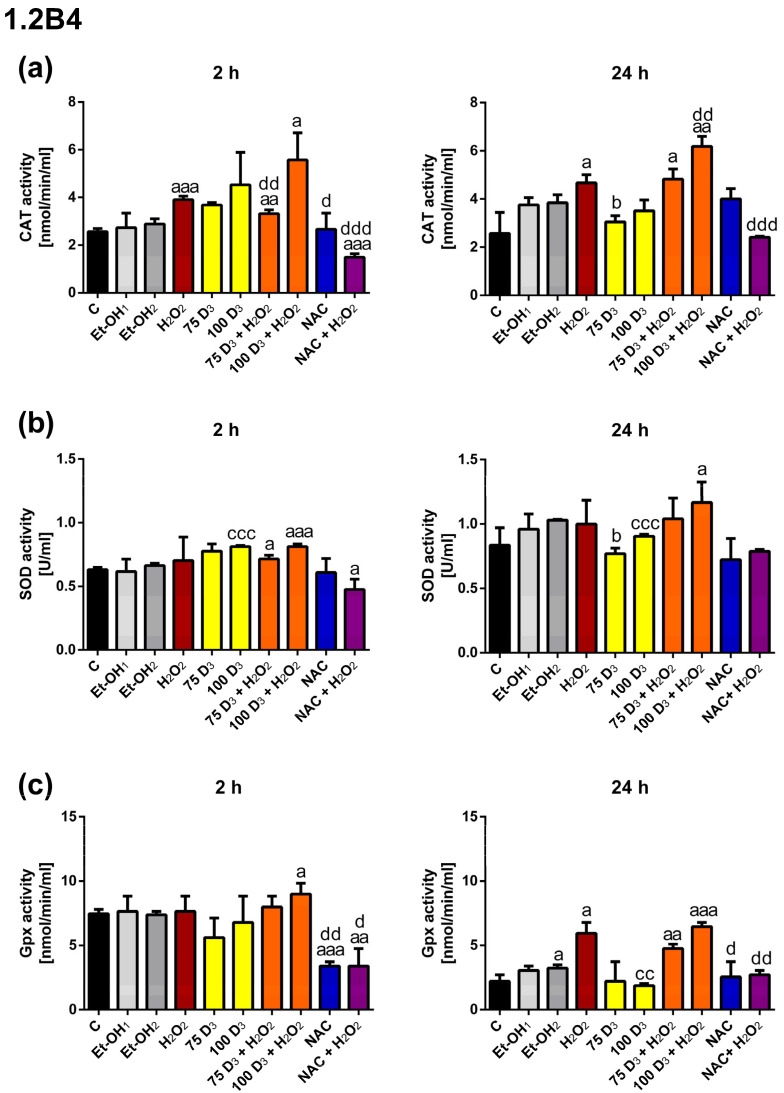
The effect of vitamin D_3_ (Vit. D_3_), hydrogen peroxide (H_2_O_2_), N-Acetyl-L-Cysteine (NAC), the combination of Vit. D_3_ with H_2_O_2_ (Vit. D_3_ + H_2_O_2_) and the combination of NAC with H_2_O_2_ (NAC + H_2_O_2_) on the activity of the antioxidant enzymes catalase (CAT) (**a**), sodium dismutase (SOD) (**b**) and glutathione peroxidase (Gpx) (**c**) in 1.2B4 cells. The cells were non-treated (C, black bars) and treated with ethanol (Et-OH, gray bars), Vit D_3_ (75 nM; 100 nM, yellow bars), H_2_O_2_ (400 µM, brown bars), Vit. D_3_ (75 nM; 100 nM) with H_2_O_2_ (400 µM, orange bars), NAC (3 mM, blue bars) and NAC (3 mM) with H_2_O_2_ (400 µM, violet bars) for 2 and 24 h. After the completion of the treatment, the cells were homogenized in cold buffer to determine CAT, SOD and Gpx activity, respectively. The absorbance was measured at 540 nm for CAT, 450 nm for SOD and 340 nm for Gpx. The data are presented as the mean ± standard deviation (SD) of the three independent experiments. ^a^ *p* < 0.05; ^aa^ *p* < 0.01; ^aaa^ *p* < 0.001 vs. C; ^b^ *p* < 0.05 vs. Et-OH_1_; ^cc^ *p* < 0.01; ^ccc^ *p* < 0.001 vs. Et-OH_2_; ^d^ *p* < 0.05; ^dd^ *p* < 0.01; ^ddd^ *p* < 0.001 vs. H_2_O_2_.

**Figure 7:**
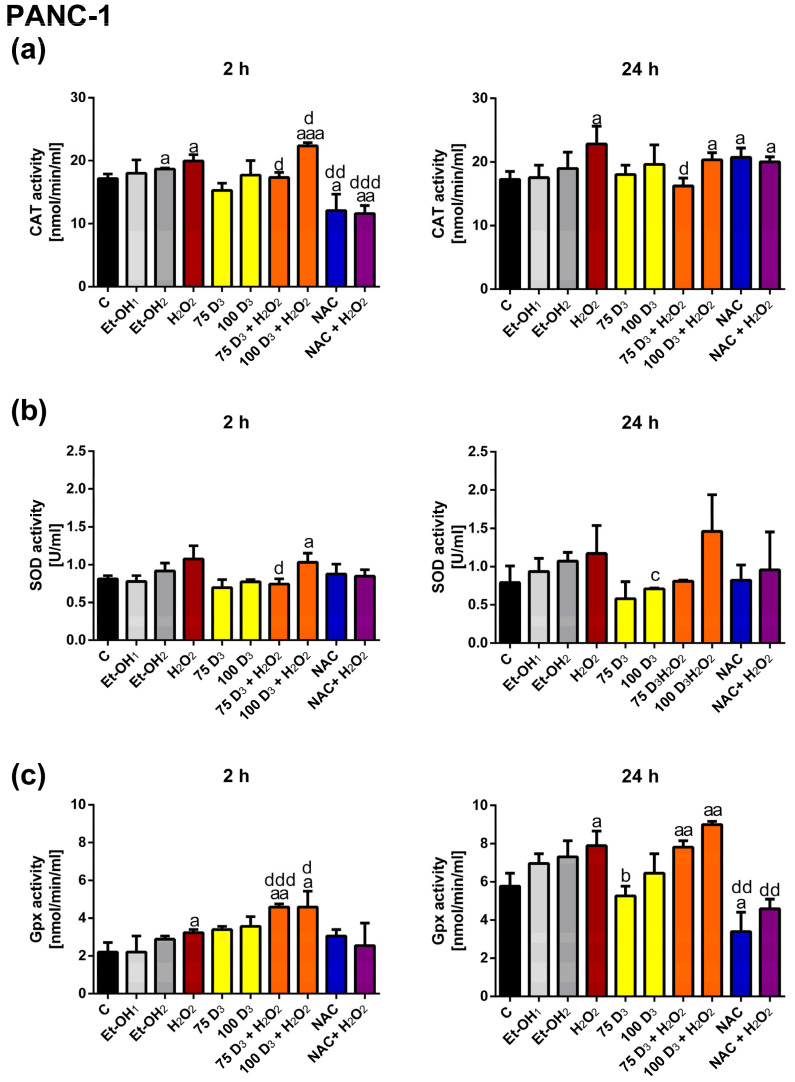
The effect of vitamin D_3_ (Vit. D_3_), hydrogen peroxide (H_2_O_2_), N-Acetyl-L-Cysteine (NAC), the combination of Vit. D_3_ with H_2_O_2_ (Vit. D_3_ + H_2_O_2_) and the combination of NAC with H_2_O_2_ (NAC + H_2_O_2_) on the activity of antioxidant enzymes catalase (CAT) (**a**), sodium dismutase (SOD) (**b**) and glutathione peroxidase (Gpx) (**c**) in PANC-1 cells. The cells were non-treated (C, black bars) and treated with ethanol (Et-OH, gray bars), Vit D_3_ (75 nM; 100 nM, yellow bars), H_2_O_2_ (400 µM, brown bars), Vit. D_3_ (75 nM; 100 nM) with H_2_O_2_ (400 µM, orange bars), NAC (3 mM, blue bars) and NAC (3 mM) with H_2_O_2_ (400 µM, violet bars) for 2 and 24 h. After the completion of the treatment, the cells were homogenized in cold buffer to determine CAT, SOD and Gpx activity, respectively. The absorbance was measured at 540 nm for CAT, 450 nm for SOD and 340 nm for Gpx. The data are presented as the mean ± standard deviation (SD) of the three independent experiments. ^a^ *p* < 0.05; ^aa^ *p* < 0.01; ^aaa^ *p* < 0.001 vs. C; ^b^ *p* < 0.05; vs. Et-OH_1_; ^c^ *p* < 0.05 vs. Et-OH_2_; ^d^ *p* < 0.05; ^dd^ *p* < 0.01; ^ddd^ *p* < 0.001 vs. H_2_O_2_.

To examine how Vit. D_3_ protects against H_2_O_2_-induced cell death, the study evaluated its antioxidant potential through the determination of ROS levels (Figure 5) and antioxidant enzymes activity (Figure 6 and Figure 7). As expected, treatment with 400 µM H_2_O_2_ for 2 and 24 h increased the level of ROS in 1.2B4 cells. In turn, Vit. D_3_ did not exert any effect on ROS generation, similarly to NAC. In 1.2B4 cells exposed to Vit. D_3_ + H_2_O_2_ the ROS level pronouncedly decreased when compared to cells treated with H_2_O_2_ for 24 h. The ROS level also diminished after 24 h of incubation with combined NAC + H_2_O_2_.

As expected, the ROS level increased in PANC-1 cells after exposure to H_2_O_2_; however, this increase was more visible after 2 h than 24 h. Vit. D_3_ and NAC did not influence the ROS level. However, when we compare the level of ROS after H_2_O_2_ treatment and the combination of Vit. D_3_ + H_2_O_2_, one can see that the Vit. D_3_ treatment markedly reduced the ROS level after 2 h, but not after 24 h. The level of ROS after exposure to NAC + H_2_O_2_ also decreased after 2 h, but surprisingly increased after 24 h.

To sum up, Vit. D_3,_ similarly to NAC, reduced the ROS level elevated by H_2_O_2_ in both 1.2B4 and PANC-1 cell lines. However, this effect was present after 24 h for 1.2B4 cells and after 2 h for PANC-1 cells. This subtle difference between 1.2 B4 and PANC-1 cells requires further exploration.

In 1.2B4 cells, H_2_O_2_ treatment for 2 h and 24 h resulted in increased CAT activity compared to the control. Vit. D_3_ and NAC alone did not affect CAT activity after 2 h and 24 h of incubation. After 2 h of incubation with 75 nM Vit. D_3_ + H_2_O_2_ as well as NAC + H_2_O_2_ we found decreased CAT activity in comparison to H_2_O_2_ exposure, whereas the incubation with 100 nM Vit. D_3_ + H_2_O_2_ evoked CAT activity. In turn, after 24 h, increased CAT activity was observed only for 100 nM Vit. D_3_ + H_2_O_2_, while NAC + H_2_O_2_ reduced CAT activity compared to H_2_O_2_ exposure.

Exposure of 1.2B4 cells to H_2_O_2_ alone, Vit. D_3_ alone and NAC alone did not significantly affect SOD activity both after 2 h and 24 h exposure (compared to the control). The treatments with Vit. D_3_ + H_2_O_2_ and NAC + H_2_O_2_ did not change SOD activity in relation to H_2_O_2_ exposure.

Gpx activity was markedly increased after 24 h treatment with H_2_O_2_ as compared to the control. The 2 h exposure to 100 nM Vit. D_3_ alone significantly increased Gpx activity, whereas NAC had the opposite effect compared to the control. Interestingly, 2 h treatemnt with NAC + H_2_O_2_ diminished Gpx activity in relation to the control. The Gpx activity was unchanged after 24 h of exposure to Vit. D_3_ + H_2_O_2_ in comparison to H_2_O_2_. Conversely, 24 h treatment with NAC + H_2_O_2_ markedly decreased Gpx activity in relation to H_2_O_2_ exposure.

In PANC-1 cells, 2 h and 24 h of H_2_O_2_ exposure increased CAT activity as compared to the control, whereas Vit. D_3_ alone did not affect CAT activity. The CAT activity after 2 h of treatment with NAC was markedly reduced, whereas after 24 h it was elevated in relation to the control. The treatment with 75 nM Vit. D_3_ + H_2_O_2_ and NAC + H_2_O_2_ for 2 h markedly decreased CAT activity as compared to H_2_O_2_. The exposure to 100 nM Vit. D_3_ + H_2_O_2_ for 2 h increased CAT activity as compared to H_2_O_2_. In turn, the treatment with 75 nM Vit. D_3_ + H_2_O_2_ significantly decreased CAT activity as compared to H_2_O_2_ after 24 h.

Similarly to 1.2B4 cells, SOD activity in PANC-1 cells was not affected by H_2_O_2_, Vit. D_3_ alone or NAC alone. The combined treatment with Vit. D_3_ + H_2_O_2_ and NAC + H_2_O_2_ did not affect SOD activity in comparison to H_2_O_2_ exposure. Only, the exposure to 75 nM Vit. D_3_ + H_2_O_2_ markedly decreased SOD activity as compared to H_2_O_2_ after 2 h.

In PANC-1 cells, H_2_O_2_ treatment for 2 h and 24 h resulted in a significant increase in Gpx activity as compared to control. Vit. D_3_ exposure for 2 and 24 h did not affect the Gpx activity as compared to the control. The 24 h treatment with NAC significantly decreased Gpx activity in relation to the control. The exposure to Vit. D_3_ + H_2_O_2_ markedly increased Gpx activity compared to H_2_O_2_ after 2 h. However, Vit. D_3_ + H_2_O_2_ incubation for 24 h did not affect the Gpx activity as compared to H_2_O_2_. Conversely, NAC + H_2_O_2_ treatment for 24 h caused a significant decrease in Gpx activity as compared to H_2_O_2_ exposure.

To sum up, H_2_O_2_-induced CAT activity was further increased by 100 nM Vit. D_3_ after 24 h which was accompanied by a reduction in ROS levels in 1.2B4 cells. The total activity of SOD and Gpx in 1.2B4 cells seemed to be unaffected by Vit. D_3._ In turn, NAC significantly abrogated H_2_O_2_-induced CAT and Gpx activity in 1.2B4 cells.

In the case of PANC-1 cells, the increases in CAT and SOD activity evoked by H_2_O_2_ were diminished by 75 nM Vit. D_3_. Interestingly, the H_2_O_2_-mediated elevations of CAT and Gpx activity were further increased by 100 nM Vit., while 75 nM Vit. D_3_ only elevated the activity of Gpx in PANC-1 cells. The action of NAC was the opposite to Vit. D_3_ since we observed that the H_2_O_2_-induced activity of CAT and Gpx was pronouncedly lowered in response to NAC. However both Vit. D_3_ and NAC were unable to efficiently decrease the level of ROS elevated by H_2_O_2_ in PANC-1 cells. NAC directly and immediately scavenges ROS and its action does not lead to the activation of antioxidant systems. In turn, Vit. D_3_ shows antioxidant action through its influence on antioxidant enzymes.

## 4. Discussion

PC, which is often diagnosed at an advanced stage, which causes a poor prognosis, is highly aggressive and resistant to treatment, which is why only 13% of patients survive for 5 years after the diagnosis [50,51]. The effect of Vit. D on the intensity of oxidative stress in cancer cells seems to be insufficiently studied. While studies on breast cancer and normal human bone cells have demonstrated that Vit. D_3_ acts as a prooxidant agent [52,53], other research involving hyperlipidemic patients with type 2 diabetes mellitus and rats with alloxan-induced diabetes have revealed its antioxidant properties [54,55]. Given these conflicting findings, the present study seeks to determine whether the reduction in oxidative stress is partially responsible for the cytoprotective effect of Vit. D_3_ in PC cells.

The first stage of the study examined the effect of H_2_O_2_ on the viability of 1.2B4 and PANC-1 cells in the presence or absence of Vit. D_3_ or NAC. It was found that H_2_O_2_ decreased the viability of both cell lines, which is consistent with previous studies showing that H_2_O_2_ reduces both cancer and normal cell viability [56,57,58,59,60,61,62,63,64]. Notably, analysis of IC_50_ values revealed that PC cells exhibit resistance to this agent. It seems that the resistance of PC cells to oxidative stress may be related to the overexpression of SOD 1–3 [44] and the fact that PC cells are resistant to damaging agents, e.g., cytostatics. Our results showed that Vit. D_3_ did not appear to influence the viability of 1.2B4 and PANC-1 cells, aligning with previous observations that low doses of Vit. D (<100 nM) do not affect the viability of cancer cells [65,66]. In contrast, some studies indicate that higher concentrations of Vit. D (>100 nM) can reduce the viability of both normal and cancer cells [67,68,69,70,71]. Regarding the effect of NAC on PC viability, our results are consistent with previous studies, showing that NAC does not influence on cell viability within the concentration range of 0.001–3 mM. Some studies also indicate that NAC may increase cell viability [72,73,74]. It is worth noting, however, that some studies have found NAC to decrease the viability of both normal and cancer cells [75,76,77,78,79].

The results of our study revealed that both Vit D and NAC reversed the toxic effect of H_2_O_2_ on PC cells. Vit. D_3_ decreased the percentage of dead 1.2B4 and PANC-1 cells in a time- and dose-dependent manner following H_2_O_2_ treatment. Similarly, a previous study found that the viability of human retinal pigment epithelial cells (ARPE-19) markedly increased after exposure to both H_2_O_2_ and 50 nM Vit. D_3_ compared to cells treated with H_2_O_2_ alone [71]. Likewise, Tohari et al. observed that low doses of Vit. D_3_ notably increased the viability of mouse cone 661W cells compared to H_2_O_2_ alone, while higher concentrations of Vit. D_3_ decreased cell viability [64]. However, another study found calcitriol and its analogs enhanced the cytotoxic effect of H_2_O_2_ on human keratinocyte cells (HaCaT) [80]. The results of our series of experiments showed that NAC protects PC cells from the cytotoxic effects of H_2_O_2_. These observations are consistent with previous studies indicating that NAC reduces the cytotoxic effect of H_2_O_2_ on normal cells [81,82,83,84,85,86]. Taken together, our results suggest that in PC cells, Vit. D_3_ possesses cytoprotective potential against H_2_O_2_-induced oxidative stress, in a similar manner to NAC.

The further step of our study evaluated the effect of Vit. D_3_ on DNA damage induced by H_2_O_2_ in PC cells. While H_2_O_2_ increased DNA damage, Vit. D_3_ was found to reduce it, though the effect was only significant after 72 h. The beneficial effect of NAC was observed already after 24 h and lasted for 72 h. These findings suggest that Vit. D_3_ employs mechanisms distinct from those of NAC. Vit. D_3_ may protect against the ROS generation caused by H_2_O_2_ and further reduce oxidative stress-induced DNA damage by enhancing the antioxidant defense system [87,88]. It has been noted that Vit. D increases the expression of NRF-2 and Klotho, which contributes to the upregulation of antioxidant enzyme gene expression [87,89,90]. Klotho elevates the expression of antioxidant enzymes such as CAT, peroxiredoxin (PRX)-2, PRX-3, SOD2 and thioredoxin reductase 1 (Trxrd-1). In turn, NRF2 upregulates CAT, Gpx, glutathione reductase (GR), SOD ½, thioredoxin (TRX) and thioredoxin reductase (TR) [87]. Klotho has also been observed to activate NRF2, leading to a decrease in oxidative damage [91]. Indeed, DNA damage caused by oxidative stress has been shown to increase in the colonic epithelial cells of mice lacking a VDR [92]. It was also found that the level of oxidative DNA damage was pronouncedly decreased in normal colorectal mucosa of patients supplemented with 800 IU of Vit. D per day [93]. Wenclewska et al. showed that supplementation with 2000 IU Vit. D per day for three months markedly reduced the level of DNA damage in the lymphocytes of subjects with dysglycemia and/or dyslipidemia; furthermore, the level of oxidative DNA damage was also diminished in the subgroup with T2DM [94]. Graziano et al. have proposed that the Vit. D–Vit. D receptor axis regulates DNA repair during oncogene-induced senescence [95]. It should be further investigated whether the reduction in H_2_O_2_-induced DNA damage by Vit. D_3_ may result from its effect on the expression of DNA repair genes, particularly those involved in homologous recombination (HR) and non-homologous end-joining (NHEJ), which remove oxidative stress-induced DNA damage. To conclude, Vit. D presents different mechanism of protection against the formation and accumulation of DNA damage than NAC. Since Vit. D enhances antioxidant defense and DNA repair—which are multi-stage processes that require more time [96]—the reduction of H_2_O_2_-induced DNA damage by Vit. D is only visible after 72 h.

In the next stage of the study, we assessed the effect of Vit. D_3_ on mRNA expression of *CAT, SOD 1–3* and *Gpx 3* in PC cells exposed to H_2_O_2_. Little is known about the effect of Vit. D on the expression of genes encoding antioxidant enzymes in PC cells. Our results indicate that H_2_O_2_ decreased the expression of antioxidant enzymes, while Vit. D_3_ enhanced their expression alone or in combination with H_2_O_2_. Lisse et al. reported a decrease in *CAT* and *SOD1* mRNA expression and an increase in *SOD2* mRNA expression after 24 h of incubation with 10 nM Vit. D in human MG-63 osteosarcoma cells. They also noted an eightfold increase in *SOD2* mRNA expression after 48 h of incubation with Vit. D_3_ [68]. Interestingly, it has also been shown that 1,25(OH)_2_D_3_ or its analogs induce the expression of thioredoxin reductase 1 (*TXNRD1*). TXNRD1 is an enzyme that converts thioredoxin into its reduced form, which has antioxidant activity [97,98]. Treatment with 50 nM 1,25(OH)_2_D_3_ increased the production of *SOD1* and *SOD2* in prostate epithelial cells (PEC) and androgen-sensitive prostate cancer cells (LNCaP) [97,99]. Therefore, the effect of Vit D_3_ on antioxidant mRNA expression seems to be dependent on its concentration. The cytoprotective action of Vit. D_3_ is associated with the expression of antioxidant defense genes. Furthermore, our data shows that the protective effect of NAC against oxidative stress is only weakly connected with the mRNA expression of antioxidant genes, highlighting a difference in the mechanism of action between Vit D_3_ and NAC. Berridge et al., found that the genomic action of Vit. D_3_ has been linked to the induction of *NFR2* mRNA expression. NFR2 is a key transcription factor that acts as a master regulator of antioxidant enzyme expression; it is possible that Vit. D may support the antioxidant defense system by targeting NRF2 [87].

It should be mentioned that the mRNA expression of antioxidant enzymes may also be controlled by numerous microRNAs, such as hsa-miR-181b-5p, hsa-miR-155-5p, hsa-miR-342-3p, hsa-miR-106b-5p, hsa-miR-92b-3p, hsa-miR-505-5p, hsa-miR-30a-5p, hsa-miR-181c-3p and hsa-miR-634 [100]. Zhao et al. concluded not only that microRNAs influence Vit. D signaling, but also that Vit. D regulates microRNA networks [101]. To the best of our knowledge, there is currently no data reporting how Vit. D_3_ regulates the expression of microRNAs targeting particular antioxidant enzymes. Upregulation of miR-200a induces nuclear translocation of the NRF2 signaling pathway, contributing to a decrease in ROS production and apoptosis of primary human osteoblasts [102]. Moreover, miR200a inhibits *KEAP1*, which leads to the stimulation of *NRF2* [103]. Under physiological conditions, KEAP1 binds to the NRF2-ECH2 homologous domain (Neh2), which suppresses NRF2 translocation to the nucleus [104]. However, under oxidative stress, KEAP1 does not interact with NRF2, triggering its separation from NRF2. Then, NRF2 translocates to the nucleus, where it binds to the antioxidant response element and stimulates the expression of antioxidant genes [105,106]. However, the influence of Vit. D on miR200a expression is not yet known.

Since we found that Vit. D_3_ affects the expression of antioxidant enzyme genes, we decided to perform an analysis of the protein levels of the tested antioxidant enzymes. We observed that the levels of these antioxidant enzymes were merely decreased after exposure to H_2_O_2._ Treatment with H_2_O_2_ in the presence of Vit. D_3_ or NAC did not exert a significant impact on the protein expression of the studied antioxidant proteins in PC cells. We only observed an increase in CAT protein expression in response to the 75 nM D_3_ + H_2_O_2_ treatment after 72 h in 1.2B4 cells. Data from the literature on the influence of Vit. D_3_ on the protein expression of antioxidant enzymes in PC cells is scarce. However, Middleton et al. report that calcitriol (10^−7^ and 10^−9^ M) and its analog seocalcitol (10^−9^ M) increased CAT protein expression in canine bladder transitional cell carcinoma (cbTCC), with no effect on SOD2 or SOD1 protein expression [107].

The next part of the study tested the effect of Vit. D_3_ on the ROS level after exposure to H_2_O_2_. As expected, H_2_O_2_ increased the ROS level, while combined incubation with H_2_O_2_ and Vit. D_3_ or NAC lowered it. These findings suggest that the cytoprotective effect of Vit. D_3_ might be a result of its antioxidant properties. We also observed that the antioxidant action of Vit. D_3_ depended on its concentration and exposure time. Consistent with our results, Tohari et al. observed that the combination of Vit. D_3_ with H_2_O_2_ reduced the ROS level by 31% after 6 h and 41% after 24 h [64]. It was also reported that administration of calcitriol to rats was associated with a significant reduction in the levels of malondialdehyde (MDA), a well-known oxidative stress marker, and the end product of lipid peroxidation [108].

Finally, we assessed how Vit. D_3_ affects the activity of antioxidant enzymes in the presence or absence of oxidative stress. It can be observed that Vit. D_3_ elevated the activity of CAT after 24 h, which was accompanied by the reduction in the H_2_O_2_-induced level of ROS in 1.2B4 cells. We also reported an increase in CAT protein expression in response to 72 h of treatment with 75 nM D_3_ + H_2_O_2_ in 1.2B4 cells. In turn, in PANC-1 cells, Vit. D_3_ elevated the activity of CAT and Gpx, which contributed to a decrease in the ROS level after 2 h. These results are consistent with findings from experiments investigating DNA damage and the mRNA expression of antioxidant enzymes. We did not show any significant effect of Vit. D_3_ on SOD activity in the presence of oxidative stress. Only a few studies have investigated the effect of Vit. D_3_ on the activity of antioxidant enzymes. In contrast to our results, AlJorhi et al. observed reduced activity of CAT after the exposure of primary cortical neuronal cells to H_2_O_2_ (0.5 mM) with a high dose of Vit. D_3_ (250 ng/mL), compared to H_2_O_2_ alone. Thus, Vit. D_3_ protected neurons against H_2_O_2-_induced oxidative stress, which was accompanied by a decrease in CAT activity [109]. Bhat et al. have demonstrated that Vit. D deficiency contributes to mild oxidative stress, which is accompanied by an increase in the activities of glutathione-dependent enzymes and a decrease in SOD and CAT activities in rat muscles. In turn, supplementation with Vit. D reversed Vit. D deficiency-related changes in all of the antioxidant enzymes tested [110]. Interestingly, Saif-Elnasr et al. did not observe any statistically significant correlation between serum Vit. D levels and the activity of SOD and GPx in T2DM patients and healthy controls. Therefore, they concluded that Vit. D supplementation does not correlate with the activity of antioxidant enzymes [111].

To conclude, H_2_O_2_-induced CAT activity was further increased by 100 nM Vit. D_3_ after 24 h, which was accompanied by a decrease in ROS levels in 1.2B4 cells. Of note, the result of Western blotting analysis also showed an elevation in CAT protein expression in response to the 75D_3_ + H_2_O_2_ treatment after 72 h in 1.2B4 cells. Vit. D_3_ did not affect the total activity of SOD and Gpx in 1.2 B4 cells. NAC markedly reduced the total activity of CAT and Gpx induced by H_2_O_2_ in 1.2B4 cells. The effect of NAC on antioxidant enzyme activity was opposite to that of Vit. D_3_ since we reported that a H_2_O_2_-induced elevation in the activity of CAT and Gpx was significantly abrogated. However, neither Vit. D_3_ nor NAC efficiently decreased the level of ROS elevated by H_2_O_2_ in PANC-1 cells. Interestingly, the results of some studies indicate that higher calcium levels stimulate the activity of antioxidants enzymes, such as CAT [112,113]. In turn, Vit. D treatment elevates intracellular calcium levels and increases the expression of calcium channel genes [114,115]. Alatavi et al. showed that the oral administration of Vit. D with calcium to diabetic rats led to a significant increase in the activities of SOD, GPO and CAT compared with the untreated group [116]. Thus, Vit. D may stimulate the activity of antioxidant enzymes by increasing intracellular calcium levels.

Recently, the influence of Vit. D on the tumor environment has attracted the attention of researchers. It has been observed that crosstalk between the components of the tumor microenvironment and cancer cells may play an important role in the process of both the development and progression of breast cancer. The components of this tumor microenvironment include immune cells, endothelial cells, stromal cells, factors secreted by these cells and the extracellular matrix. Cancer-associated fibroblasts are the most abundant cell type in both the breast cancer and PC microenvironments [117,118]. Łabędź et al. investigated the influence of Vit. D on breast cancer-associated fibroblasts and suggested that calcitrol may alter their immunosuppressive or procancer properties. The observed anticancer polarization of these cancer-associated fibroblasts in response to treatment may be related to a reduction in CCL2, TNC, MMP9 and MMP-2. Conversely, the opposite action may be associated with elevated PDPN, TIMP1 and SPP1. However, cancer-associated fibroblast-conditioned media from nonmetastatic and postmenopausal patients incubated ex vivo with calcitriol reduced the migration of MCF-7 cells [119]. The influence of Vit. D, which has anti-inflammatory and immunomodulatory properties, on the microenvironment of PC is not yet known and is an excellent direction for further research on this tumor. Therefore, the effect of Vit. D on fibroblasts, immune cells and cancer cells in the tumor microenvironment may be different. Our study concerned only cancer cells. The obtained results showed that Vit. D protects PC cells from the harmful effects of H_2_O_2_. However, at this stage, it remains unclear how cancer cells communicate with normal cells within the tumor. We also do not know what effect Vit. D would have in the tumor on normal cells such as fibroblasts or immune cells.

The results of clinical trials are still inconclusive; it is not clear whether patients with PC can benefit from Vit. D supplementation or not. Interestingly, Wang et al. reported that VDR is highly expressed in PC cells [37]. Moreover, it was also suggested that the expression level of VDR may be a potential prognostic factor for PC patients [37]. A recent study carried out by Li et al. showed that PC patients with VDR overexpression in pancreatic tumor cells had significantly decreased overall survival. This overexpression of VDR in PC cells promotes the recruitment and polarization of macrophages into M2 macrophage phenotype via the secretion of CCL20, which leads to the activation of tumor progression [120]. Further clinical trials based on combined therapies using Vit. D are necessary in PC patients, both with and without overexpressed VDR in PC cells, to observe their impact on survival/extension of life and the delay of further progression of PC.

It should also be noted that our study has several limitations. Firstly, it would be beneficial to determine the level of oxidative DNA damage using a comet assay with formamidopyrimidine DNA glycosylase (Fpg) and endonuclease III (Nth). Secondly, the PANC-1 and 1.2B4 cell lines have different origins, which may be responsible for some of the variation observed in the results. PANC-1 is a pancreatic adenocarcinoma [121], whereas 1.2B4 is a human hybrid cell line of HuP3 (adenocarcinoma) cells and the primary culture of human pancreatic islets. Thirdly, while the action of Vit. D seems to be well-characterized in numerous normal cell lines, its effects on normal pancreatic duct cells, the origin of most pancreatic adenocarcinomas, remain poorly understood. Fourth, it is also important to note that the MTT assay used for assessing cell viability is non-specific. Therefore, the increase in formazan production observed at NAC concentrations above 3 mM may result from factors other than increased cell viability, such as enhanced cell growth or mitochondrial activity.

To summarize, our series of experiments showed that PC cells are resistant to DNA damaging effects caused by a high concentration of H_2_O_2_. Moreover, the results obtained indicate that Vit. D_3_ protects PC cells against death induced by H_2_O_2_. This effect was accompanied by a reduction in ROS level and DNA damage, as well as the mRNA upregulation of CAT, SOD 1-3 and Gpx3. Interestingly, 100 nM Vit. D_3_ increased, while 75 nM Vit. D_3_ reduced, the activity of CAT in 1.2B4 and PANC-1 cells, as well as SOD in PANC-1 cells exposed to H_2_O_2_-induced oxidative stress. Our findings suggest that Vit. D_3_ may exert antioxidant activity but is not a free radical scavenger like NAC. However, other possible molecular mechanisms should also be explored to identify how Vit. D_3_ supports PC cell viability and protects against DNA damage induced by oxidative stress.

## Data Availability

Data is contained within the article.

## Supplementary Materials

The following supporting information can be downloaded at: https://www.mdpi.com/article/10.3390/antiox14091101/s1.
