# Supplementary material for: Vitamin D Protects Pancreatic Cancer (PC) Cells from Death and DNA Damage Induced by Oxidative Stress
Source: Antioxidants (Basel). 2025 Sep 10;14(9):1101. doi: 10.3390/antiox14091101 (PMC12466453; doi:10.3390/antiox14091101)
Supplement: Supplementary file 1 [file antioxidants-14-01101-s001.zip › antioxidants-3719641-supplementary.pdf]

(a) 1.2B4

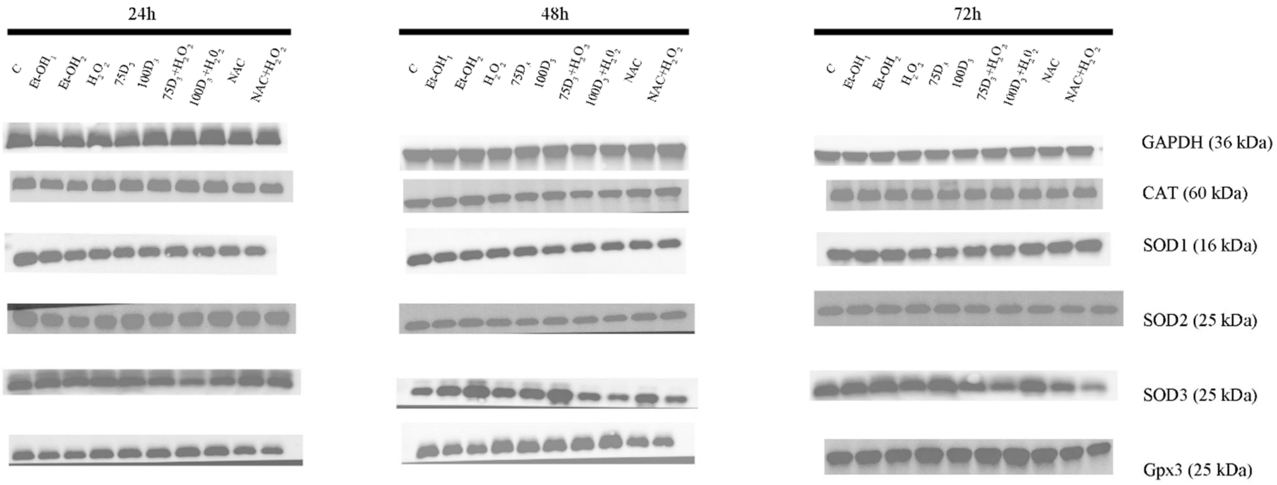
24h
48h
72h
C
Et-OH1
Et-OH2
H2O2
75D3
100D3
75D3+H2O2
100D3+H2O2
NAC
NAC+H2O2
GAPDH (36 kDa)
CAT (60 kDa)
SOD1 (16 kDa)
SOD2 (25 kDa)
SOD3 (25 kDa)
Gpx3 (25 kDa)

1.2B4

(b)

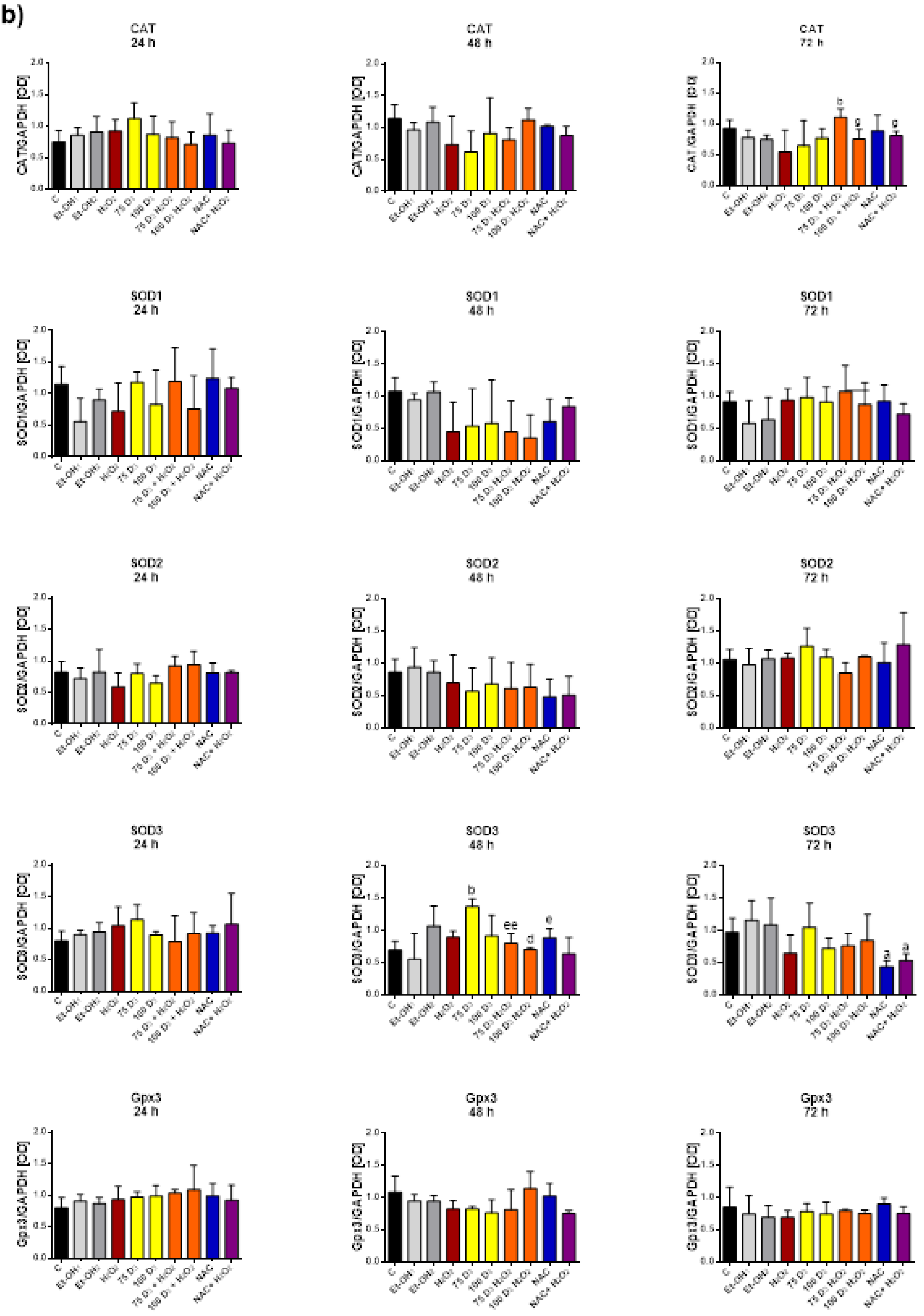

Figure S1. The effect of vitamin D_3 (Vit. D_3), hydrogen peroxide (H_2O_2), N-Acetyl-L-Cysteine (NAC), combinations of Vit. D_3 with H_2O_2 (Vit. D_3, + H_2O_2) and NAC with H_2O_2 (NAC + H_2O_2) on protein expression of antioxidant enzymes: catalase (CAT), sodium dismutase 1 (SOD1), sodium dismutase 2 (SOD2), sodium dismutase 3 (SOD3), glutathione peroxidase 3 (Gpx3) in 1.2B4 cells. The expression of

proteins in 1.2B4 cells ((a) representative blots and (b) quantitative graphs) was determined by Western blotting method. The 1.2B4 cells were non-treated (C, black bars) and treated with ethanol (Et-OH, grey bars), Vit D_3 (75 nM; 100 nM, yellow bars), H_2O_2 (400 µM, brown bars), Vit. D_3 (75 nM; 100 nM) with H_2O_2 (400 µM, orange bars), NAC (3 mM, blue bars), NAC (3 mM) with H_2O_2 (400 µM, violet bars) for 24, 48, and 72 h. The data are expressed as a percentage of optical density (OD) over the background ratio of target protein expression in relation to GAPDH. Data are presented as OD means ± standard deviation (SD). $^a p < 0.05$ *vs.* C; $^b p < 0.05$ *vs.* Et-OH_1; $^d p < 0.05$ *vs.* H_2O_2; $^e p < 0.05$; $^{ee} p < 0.01$ *vs.* $75D_3$; $^g p < 0.05$ *vs.* $75D_3+H_2O_2$.

(a) PANC-1

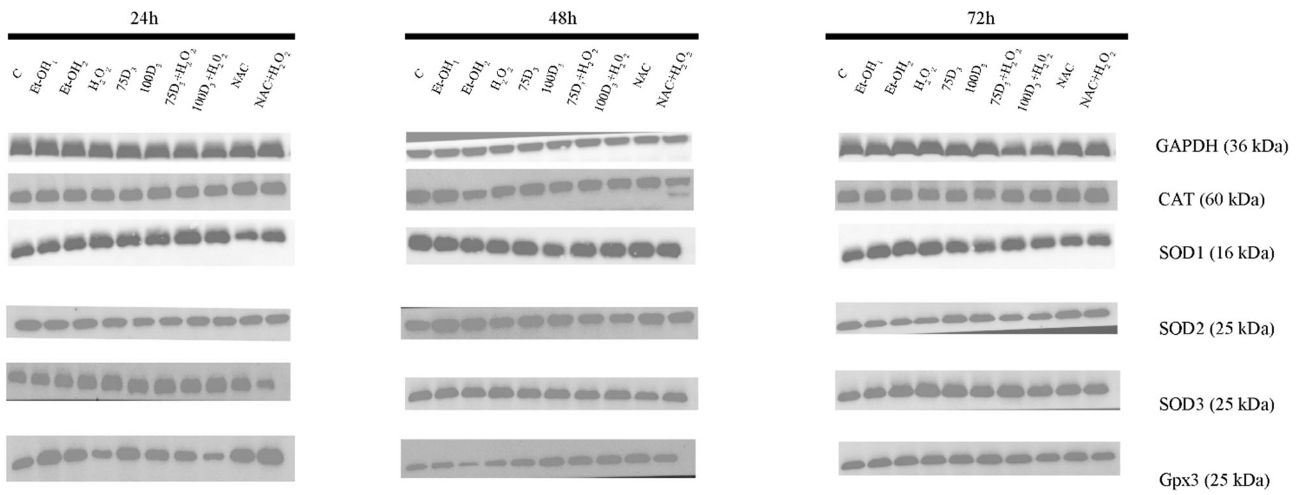

PANC-1

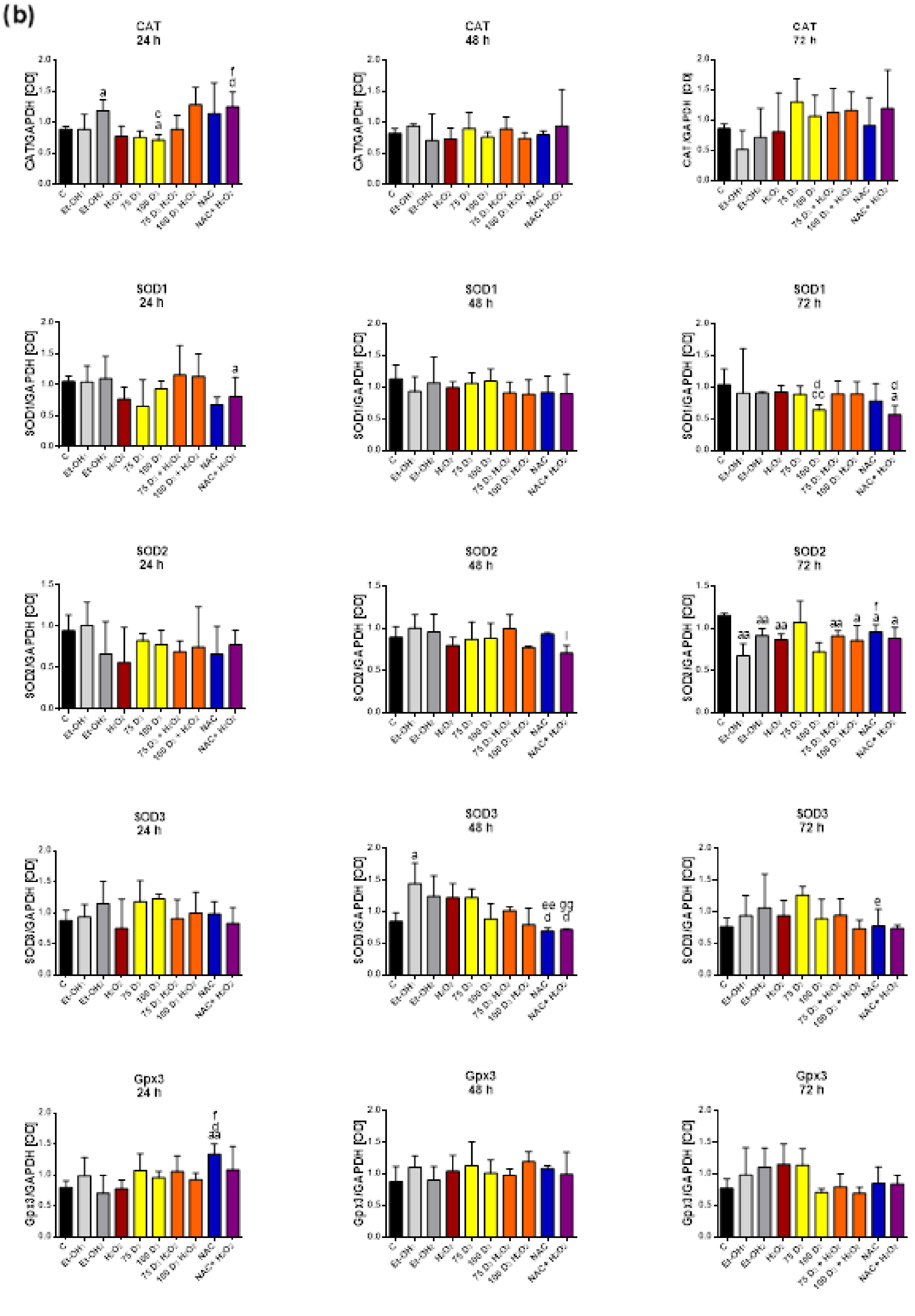

Figure S2. The effect of vitamin D_3 (Vit. D_3), hydrogen peroxide (H_2O_2), N-Acetyl-L-Cysteine (NAC), combination of Vit. D_3 with H_2O_2 (Vit. D_3, + H_2O_2) and NAC with H_2O_2 (NAC + H_2O_2) on protein expression of antioxidant enzymes: catalase (CAT), sodium dismutase 1 (SOD1), sodium dismutase 2

(SOD2), sodium dismutase 3 (SOD3), glutathione peroxidase 3 (Gpx3) in PANC-1 cells. The expression of proteins in PANC-1 cells ((a) representative blots and (b) quantitative graphs) was determined by Western blotting method. The PANC-1 cells were non-treated (C, black bars) and treated with ethanol (Et-OH, grey bars), Vit D_3 (75 nM; 100 nM, yellow bars), H_2O_2 (300 µM, brown bars), Vit. D_3 (75 nM; 100 nM) with H_2O_2 (300 µM, orange bars), NAC (3 mM, blue bars), NAC (3 mM) with H_2O_2 (300, violet bars) for 24, 48, and 72 h. The data are expressed as a percentage of optical density (OD) over the background ratio of target protein expression in relation to GAPDH. Data are presented as OD means ± standard deviation (SD). $^{a}p < 0.05$; $^{aa}p < 0.01$ *vs.* C; $^{c}p < 0.05$; $^{cc}p < 0.01$ *vs.* Et-OH_2; $^{d}p < 0.05$ *vs.* H_2O_2; $^{e}p < 0.05$; $^{ee}p < 0.01$ *vs.* $75D_3$; $^{f}p < 0.05$ *vs.* $100D_3$; $^{gg}p < 0.01$ *vs.* $7D_3+H_2O_2$; $^{i}p < 0.05$ *vs.* NAC.

(a) 1.2B4

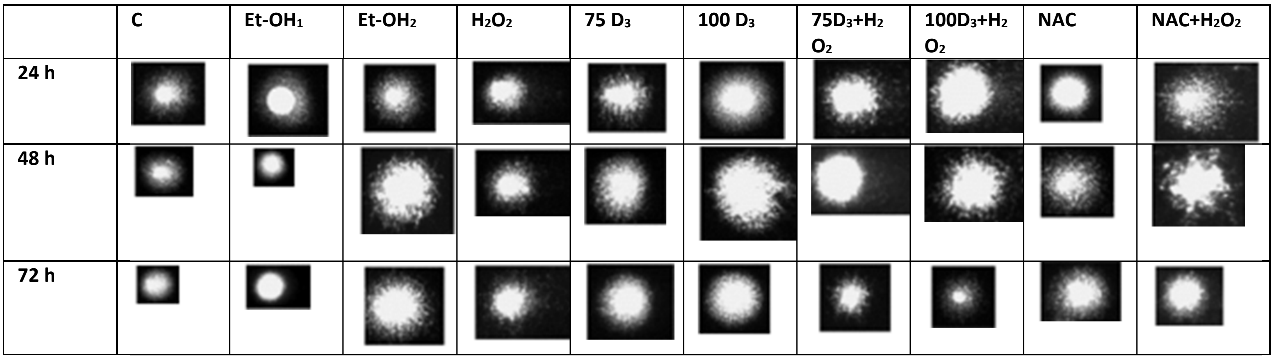

(b) PANC-1

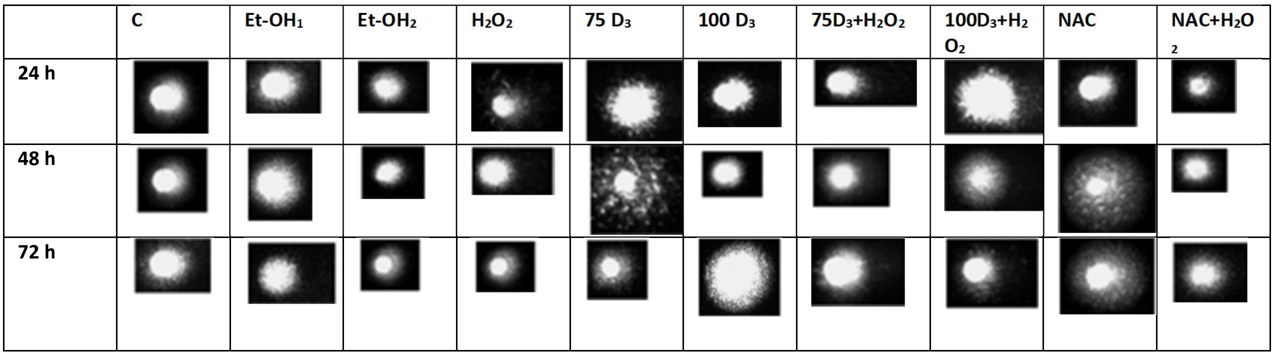

Figure S3. The effect of vitamin D_3 (Vit. D_3), hydrogen peroxide (H_2O_2), N-Acetyl-L-Cysteine -acetylcysteine (NAC), combination of Vit. D_3 with H_2O_2 (Vit. D_3, + H_2O_2), and NAC with H_2O_2 (NAC + H_2O_2) on DNA damage level in pancreatic cancer (PC) cells. Representative photos for 1.2B4 cells (a) and PANC-1 cells (b).
